# Actin dynamics and the Bmp pathway drive apical extrusion of proepicardial cells

**DOI:** 10.1242/dev.174961

**Published:** 2019-07-04

**Authors:** Laura Andrés-Delgado, Alexander Ernst, María Galardi-Castilla, David Bazaga, Marina Peralta, Juliane Münch, Juan M. González-Rosa, Inês Marques, Federico Tessadori, José Luis de la Pompa, Julien Vermot, Nadia Mercader

**Affiliations:** 1 Development of the Epicardium and its Role During Regeneration Laboratory, Centro Nacional de Investigaciones Cardiovasculares Carlos III (CNIC), Melchor Fernández Almagro 3, 28029 Madrid, Spain; 2 Department of Anatomy, Histology and Neuroscience, School of Medicine, Universidad Autónoma de Madrid, 28029 Madrid, Spain; 3 Institute of Anatomy, University of Bern, 3000 Bern 9, Switzerland; 4 Institut de Génétique et de Biologie Moléculaire et Cellulaire, 67404 Illkirch, France; 5 Centre National de la Recherche Scientifique, UMR7104, 67404 Illkirch, France; 6 Institut National de la Santé et de la Recherche Médicale, U964, 67404 Illkirch, France; 7 Université de Strasbourg, 67411 Illkirch, France; 8 Intercellular Signaling in Cardiovascular Development and Disease Laboratory, Centro Nacional de Investigaciones Cardiovasculares Carlos III (CNIC), Melchor Fernández Almagro 3, 28029 Madrid, Spain; 9 Ciber CV, 28029 Madrid, Spain; 10 Hubrecht Institute-KNAW and UMC Utrecht, Uppsalalaan 8, 3584CT Utrecht, The Netherlands

**Keywords:** Actomyosin, Bmp, Cell extrusion, Proepicardium, Zebrafish, Heart development

## Abstract

The epicardium, the outer mesothelial layer enclosing the myocardium, plays key roles in heart development and regeneration. During embryogenesis, the epicardium arises from the proepicardium (PE), a cell cluster that appears in the dorsal pericardium (DP) close to the venous pole of the heart. Little is known about how the PE emerges from the pericardial mesothelium. Using a zebrafish model and a combination of genetic tools, pharmacological agents and quantitative *in vivo* imaging, we reveal that a coordinated collective movement of DP cells drives PE formation. We found that Bmp signaling and the actomyosin cytoskeleton promote constriction of the DP, which enables PE cells to extrude apically. We provide evidence that cell extrusion, which has been described in the elimination of unfit cells from epithelia and the emergence of hematopoietic stem cells, is also a mechanism for PE cells to exit an organized mesothelium and fulfil their developmental fate to form a new tissue layer, the epicardium.

## Abstract (precis)

**Summary:** Proepicardial cells emerge from the pericardial mesothelium through apical extrusion, a process that depends on BMP signaling and actomyosin rearrangements.

## INTRODUCTION

The epicardium is the outer mesothelial layer of the heart. During development, the epicardium sustains the underlying myocardium through paracrine signals that promote its growth (Olivey and Svensson, 2010; Pérez-Pomares and de la Pompa, 2011). It is also an important cell source during embryogenesis. Epicardium-derived cells (EPDCs) differentiate into cardiac fibroblasts and other cell types (Chau et al., 2014). Upon cardiac injury in zebrafish, EPDCs promote tissue repair and regeneration (Kennedy-Lydon and Rosenthal, 2015; Simoes and Riley, 2018).

The epicardium derives from the proepicardium (PE), a cluster of cells that emerges from the pericardium close to the venous pole (VP) of the heart tube during heart looping and after the onset of heart beating (Maya-Ramos et al., 2013; Schulte et al., 2007). In zebrafish, PE formation is regulated by bone morphogenetic protein (Bmp) signaling (Liu and Stainier, 2010). Accordingly, mutants for the Bmp receptor Acvr1l do not form a PE, whereas *bmp2b* overexpression expands PE marker gene expression (Liu and Stainier, 2010)*.* During PE formation, cells change their polarity, suggesting that an epithelial–mesenchymal-like transition has a role in cluster generation (Hirose et al., 2006; Serluca, 2008; Tandon et al., 2016; Wu et al., 2010).

Once the PE forms, the heartbeat has an essential role in allowing PE cells to be ‘washed away’ into the pericardial cavity. The heartbeat generates a pericardial fluid flow, allowing the PE cells to detach from the mesothelium. Floating PE cells adhere to the myocardial surface, and ultimately spread over the surface to form the epicardium (Peralta et al., 2013; Plavicki et al., 2014).

During morphogenesis, cell migration and proliferation result in the continuous rearrangement of mechanical properties of tissue layers. Collective cell migration and proliferation can lead to local cell crowding and the generation of tissue tension (Eisenhoffer et al., 2012; Tada and Heisenberg, 2012). Additionally, changes in tissue growth can further influence cell signaling (Aegerter-Wilmsen et al., 2012; Hiscock and Megason, 2015). The actomyosin cytoskeleton plays a central role in controlling cell shape and developmental events (Heisenberg and Bellaiche, 2013; Levayer and Lecuit, 2012; Martin et al., 2009; Munjal and Lecuit, 2014). It is tightly associated with membrane junction complexes and can react to extracellular signals or signals from neighboring cells by altering cell properties (Lecuit and Yap, 2015; Martin et al., 2010; Munjal and Lecuit, 2014).

The PE comprises mesothelial cells from the dorsal pericardium (DP). Mesothelia share some commonalities with epithelia and it is therefore interesting to draw parallels to learn more about their development and homeostasis. Epithelial layers are maintained by cell division, intercalation and extrusion (Guillot and Lecuit, 2013), which are interconnected; for instance, cell proliferation is also a major driver of cellular intercalation and thus tissue organization in growing embryos (Firmino et al., 2016). Cell extrusion in epithelia is often observed during morphogenesis, including tissue folding (Ambrosini et al., 2017; Monier et al., 2015; Saias et al., 2015), and the emergence of hematopoietic cells (Kissa and Herbomel, 2010; Lancino et al., 2018). It remains unclear how canonical developmental signaling pathways can influence these cellular behaviors and whether extrusion can also be a morphogenetic event occurring in mesothelia.

Here, we used zebrafish to study the morphogenetic events leading to PE formation. We found that cells from the DP collectively move towards the DP midline, where some of them round up and extrude into the pericardial cavity to form the PE cluster. These processes depend on Bmp signaling, which regulates actomyosin dynamics. Our results reveal how signaling molecules influence morphogenesis and show that PE formation relies on complex tissue rearrangements within the pericardial mesothelium.

## RESULTS

### Constriction of the dorsal pericardium leads to apical extrusion of proepicardial cells

**Fig. 1.**
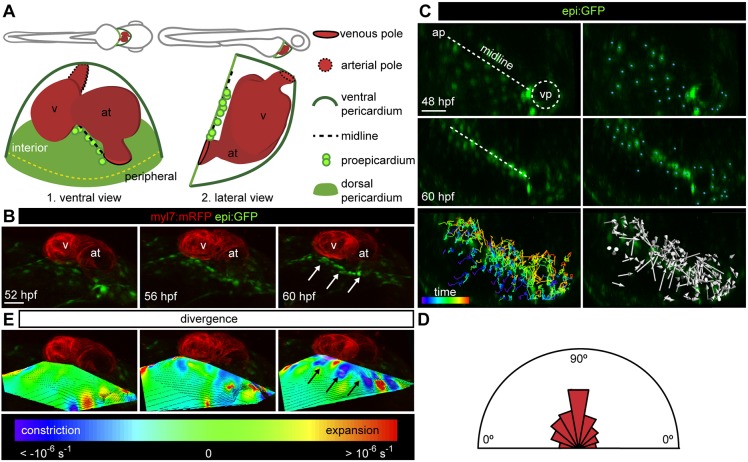
**Constriction of the dorsal pericardium precedes proepicardium formation.** (A) Scheme of lateral and ventral views of a 60 hpf zebrafish embryo (top) and heart (bottom). The lateral view is rotated by −90°. (B) 3D ventral view of heart region in an *epi:GFP; myl7:mRFP* embryo. *epi:GFP*^+^ cells (green) are present in the dorsal pericardium (DP) and proepicardium (PE), *myl7:mRFP*^+^ cells (red) form the myocardium. 3D projections from *in vivo* time lapse at different time points are shown. White arrows point to PE cells (see Movie 1). (C) First and last frame of an *in vivo* time lapse of DP cells in an *epi:GFP* embryo; the midline is shown by a dashed white line, blue dots indicate tracked cells. Full colored tracks label the first time frame in purple and the last in red. Arrows indicate overall direction of tracked cells. (D) The half-rose diagram shows the movement angles of tracked cells relative to the midline. (E) 3D projection of the divergence field calculated for the time-lapse video above. The calculated divergence values are represented by colors; purple-blue regions represent constriction and red regions represent expansion. Black arrows point to PE cell clusters. ap, arterial pole; at, atrium; hpf, hours post-fertilization; v, ventricle; vp, venous pole. DP digitally isolated in 3D projections. Shown is data from one imaged animal representative of three biological replicates. Scale bars: 50 µm.

To investigate PE formation, we analyzed the movement of mesothelial cells in the pericardium of zebrafish embryos. Most PE cells appear as clusters in the DP proximal to the VP and the atrio-ventricular canal (AVC) of the heart tube (Fig. 1A). We analyzed PE formation ∼52 h post-fertilization (hpf), before the PE clusters are visible. For live imaging, we used the enhancer trap lines *Et(-26.5Hsa.WT1-gata2:EGFP)^cn1^* or *Et(-26.5Hsa.WT1-gata2:EGFP)^cn14^* (hereafter termed *epi:GFP*) in which GFP expression is controlled by the *wilms tumor 1a* (*wt1a*) regulatory elements, and recapitulates its expression pattern (Fig. 1A; Peralta et al., 2013). This allowed us to perform cell tracking, as GFP signal is present in all DP cells, particularly around the cell nucleus, and to resolve individual DP cell movements in the mesothelium (Peralta et al., 2014). We tracked DP cells using time-lapse confocal microscopy at 52-60 hpf, and observed that they converged at a region spanning from the VP to the arterial pole (AP) (Fig. 1B,C; Movie 1). This region within the DP was defined as the midline. We measured the angle of the cell trajectories within the DP in relation to the midline. The majority of cell trajectory angles were ∼90°, indicating that DP cells move nearly perpendicular to the midline (Fig. 1D; *n*=3 embryos). These data suggest that mesothelial cell movements result in an accumulation of cells at the midline where the PE cells will emerge.

To characterize the morphological changes to the DP during PE formation in *epi:GFP* embryos, we developed a method to quantify cell rearrangements in 3D tissue monolayers using a customized divergence field calculator (Fig. S1). The resulting divergence field describes a set of cell movements relative to each other. Negative divergence values indicate that cells converge and positive values that cells move away from each other. To associate this divergence with location within the DP, we overlaid the divergence field with each time point of the recordings. We used a color code, based on the calculated divergence, to indicate tissue constriction or expansion. We imaged the DP in three embryos at 43 different time points every 12 min, and measured 150 DP cell tracks. This analysis revealed that the highest local levels of constriction located at the midline, the site of PE formation (Fig. 1E). By calculating a mean divergence of −0.1±0.05 s^−1^ (mean±s.d.) we also confirmed an overall constriction of the DP from 52 to 60 hpf.

### Dorsal pericardial cells reduce their area during displacement to the midline

**Fig. 2.**
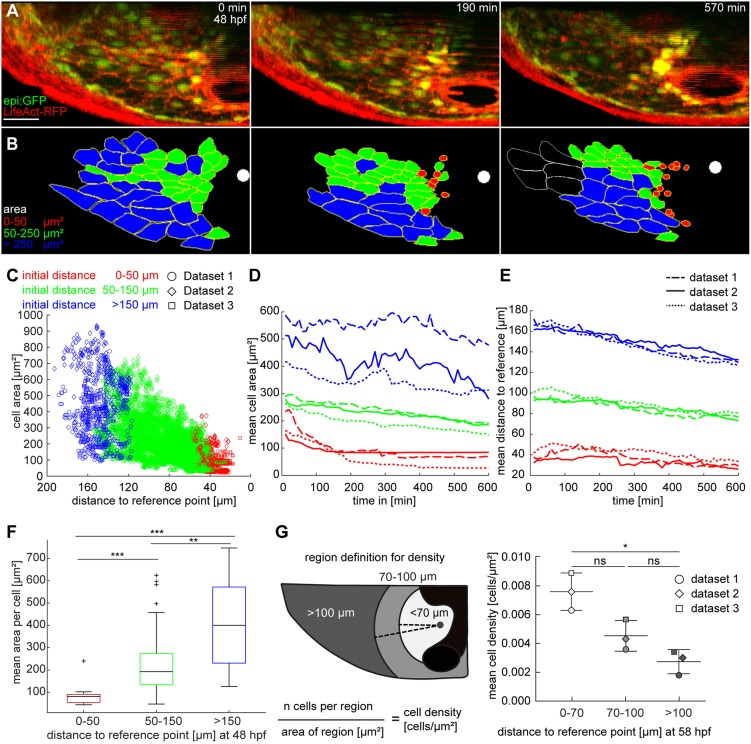
**Dorsal pericardial cells reduce their area during displacement to the midline.** (A) Maximum intensity projections from a time lapse using *epi:GFP;* *βactin:LifeAct-RFP^e2212Tg^* embryos from 48 hpf onwards. LifeAct-RFP (red) signal shows F-actin. (B) Color-coded segmentation of 47 cell shapes from time series in A. Segmentation was repeated every 10 min in 60 subsequent time frames (*n*=3 animals). White spots indicate the reference point on the midline. Last frame shows white outline for cells not included in the quantification (see Movie 2). (C) The scatter plot shows the area of each cell per time point against the distance to the reference location. Color code represents the initial distance to the reference point. The mean distance is drawn against the distance to reference. (D,E) Graphs describe tracking and analysis of each cell from three datasets, with mean cell area (D) and mean distance of each cell (E) drawn against time. Each dataset is split into three categories according to the initial distance of a cell to the reference point, indicated by the color code. The line style indicates different datasets. (F) Data are sorted into categories by the initial distance to the reference point; each category is averaged and represented in a boxplot. The box represents the 25-75th percentiles, and the median is indicated. The whiskers show minimum and maximum of observed values. Outliers are indicated by crosses. (G) At the last time point (58 hpf), the mean cell density within the DP was calculated 70 µm, 100 µm and >100 µm from the reference point. The principle of measurement is schematically represented on the left. Areas in which the signal intensity is weak owing to the heart tube (black) were excluded from the quantification. Data are means±s.d.; Kruskal–Wallis test. ns, not significant; *P<0.05; **P<0.01; ***P<0.001. hpf, hours post-fertilization. DP digitally isolated in maximum intensity projections. Data from three out of six biological replicates from two independent acquisitions. Scale bar: 50 µm.

To characterize cellular changes to DP cells during their displacement to the midline, we analyzed the DP cell area during PE formation. To locate F-actin *in vivo* (Riedl et al., 2008) we used the double transgenic line *epi:GFP; βactin:LifeAct-RFP^e2212Tg^* (hereafter termed LifeAct-RFP). The LifeAct-RFP signal was strong close to the inner cell membrane, which allowed us to trace cell shapes over time (Fig. 2A). We defined a point on the midline close to the AVC as a reference point and quantified changes in DP cell area at different distances from this reference. Cells with a larger area were further away from the reference point at the end of the time-lapse movie, whereas the smallest cells were close to the midline (Fig. 2B; Movie 2). Sorting cell areas into three categories according to their initial distance from the reference point (distance >150 µm, 50-150 µm, 50 µm) revealed that cells reduce their cell area as they get closer to the midline over time (Fig. 2C-E). In agreement, comparison of the distance categories showed a significant decrease of cell area near the reference on the midline (Fig. 2F). Quantification of the number of cells in different parts of the DP at the final time point of the time lapse revealed that the cell density was significantly higher close to the midline (<70 µm) as compared with the peripheral DP (Fig. 2G).

In sum, DP cells reduce their area during displacement towards the midline, suggesting that they concomitantly lose cell-cell contact area. This reduction in cell area at the midline might contribute to DP tissue constriction and represent a first step of PE formation.

### Dorsal pericardial cells extrude apically to form proepicardial clusters

**Fig. 3.**
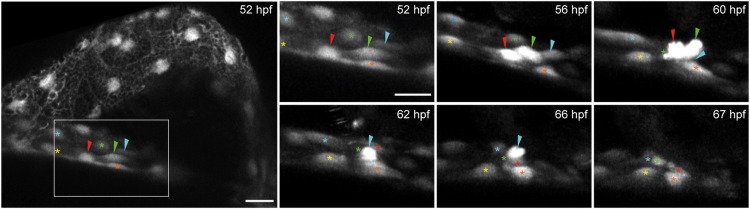
**Collective cell movements and apical extrusion lead to proepicardium delamination.** *epi:GFP* embryo *in vivo* time lapse. Maximum intensity projection of 22 µm. Left panel, overview of the pericardial cavity at 52 hpf. Right panels, zoomed views of the dorsal pericardium (DP) during proepicardium (PE) formation. Shown are frames of the time lapse from 52 to 67 hpf. Colored arrowheads, emerging PE cells. Colored asterisks, DP cells surrounding PE cells (shown is one out of 10 observed events, see also Movie 3). hpf, hours post-fertilization. Images from one out of 10 embryo from five independent acquisitions. Scale bar: 20 µm; zoomed images, 10 µm.

We next characterized the emergence of PE cells by *in vivo* time-lapse imaging of the *epi:GFP* line. During displacement, we observed that DP cells close to the midline round up (Fig. 3; Movie 3), and that the cells surrounding these emerging PE cells came closer together. Ultimately, one or multiple cells protruded from the DP layer and remained loosely attached to the neighboring DP mesothelial cells. The cells that were bordering the newly formed PE cells subsequently converged under the rounded PE cell, which extruded towards the pericardial lumen. To investigate whether DP cells show apicobasal polarity, we visualized a set of polarity-associated marker proteins: the basolateral marker β-catenin, the basally deposited extracellular matrix molecule Laminin and the apical marker Par3 (Campanale et al., 2017; Manninen, 2015) (Fig. S2). Immunostaining for β-catenin showed its localization at DP cell junctions (Fig. S2A). Staining against Laminin revealed its accumulation beneath the abluminal side of DP cells (Fig. S2B). Finally, we injected *Par3-RFP* mRNA into 1-cell stage embryos, and observed that it accumulated on the luminal DP outer membrane at 48 hpf (Fig. S2C,D). The results confirm that the apical cell membrane of DP cells faces the lumen of the pericardial cavity. Thus, PE formation occurs through the local overcrowding of cells at the DP midline, inducing extrusion of DP mesothelial cells to the apical side. This cell behavior is reminiscent of the process of apical extrusion, allowing cells to bulge out and leave organized epithelia (Eisenhoffer et al., 2012).

### Proliferation of dorsal pericardial cells contributes to cell constriction at the midline

**Fig. 4.**
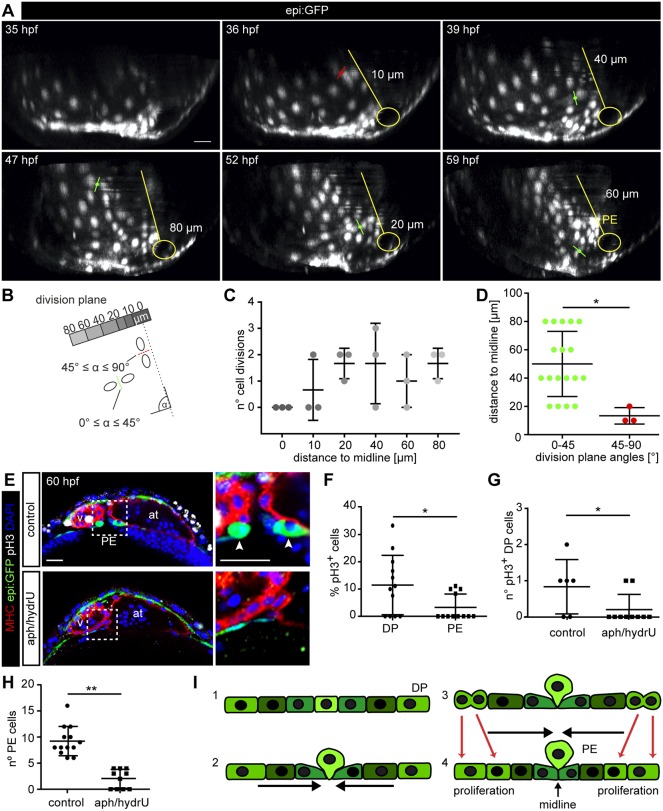
**Proliferation of dorsal pericardial cells contributes to proepicardium formation.** (A) 3D projection of six frames of an *epi:GFP* embryo *in vivo* time lapse (see also Movie 4). Division planes indicated with red (45°≤α≤90°) or green (0°≤α≤45°) lines. A representation of the midline and the venous pole is shown in yellow. The distance of the division to the midline is indicated. (B) Scheme showing the principle of measurements for distance and angles of division planes to midline. (C) Number of cell divisions during *in vivo* time-lapse imaging relative to their distance to midline (three embryos with 20 cell divisions each)*.* (D) Angles of cell division planes and their distance relative to the midline. A-D, data from ≥10 biological and ≥2 technical replicates. (E) Immunostaining for GFP (green), Myosin heavy chain (red) and pH3 (white). DAPI counterstained nuclei (blue). Optical sections of a control heart or embryos treated with aphidilcolin and hydroxyurea (aph/hydrU). Zoomed view of the PE area on the right. Arrowheads, PE cells. (F) Percentage of pH3^+^ cells in the dorsal pericardium (DP) compared with the PE in control animals. (G) Quantification of total number of pH3^+^ cells in the DP. (H) Quantification of PE cell number. (I) Scheme of PE extrusion mechanism: (1) DP is a flattened mesothelium; (2) DP cells move towards the midline; (3) PE cells round up at the midline; (4) DP cell proliferation contributes to the constriction and PE cells finally extrude. Data are mean±s.d., Kruskal–Wallis test followed by multiple comparison test in C; two-tailed Student's *t*-test in D,F-H, *P<0.05; **P<0.01. at, atrium; hpf, hours post-fertilization; PE, proepicardium; v, ventricle. DP digitally isolated in 3D projections. E-H, representative data from 10 biological replicates out of one experiment. Scale bars: 20 µm.

To determine the contribution of cell proliferation in the process of DP constriction, we analyzed the spatial distribution of cell divisions within the DP from 35 to 60 hpf. We measured the distance of the cell division plane to the midline and found that pericardial cells divide throughout the DP layer (Fig. 4A,B; Movie 4). Cell proliferation neither occurred directly at the midline nor preferentially close to it (Fig. 4C). By categorizing the orientation of cell divisions, we found that the cells divided more frequently perpendicular than parallel to the midline (*P*=0.0144), and cell divisions close to the midline were more often parallel to the midline (Fig. 4D).

We next assessed cell proliferation by immunostaining for phospho-histone 3 (pH3) on fixed *epi:GFP* embryos. While we detected proliferating cells in the pericardium (Fig. 4E,F), only one pH3^+^ PE cell was observed in four out of 12 embryos. Inhibiting cell proliferation from 48 hpf onwards with aphidilcolin/hydroxyurea (aph/hydrU), which inhibits DNA synthesis (Mutomba and Wang, 1996), significantly reduced pH3^+^ cell number in the DP at 60 hpf [from 0.8±0.3 to 0.2±0.1 (mean±s.d.); Fig. 4G]. The treatment also significantly reduced the number of PE cells (from 9±3 to 2±1; Fig. 4H). We conclude that cell proliferation within the PE is not a driving force of PE emergence. Instead, cell division in the DP is involved in PE formation. Proliferation might lead to an increase in DP cell number, contributing to the local crowding of DP cells at the midline, which ultimately leads to PE cluster formation (Fig. 4I).

### Heartbeat-induced pericardial fluid advections are dispensable for proepicardium extrusion, but influence proepicardial localization

**Fig. 5.**
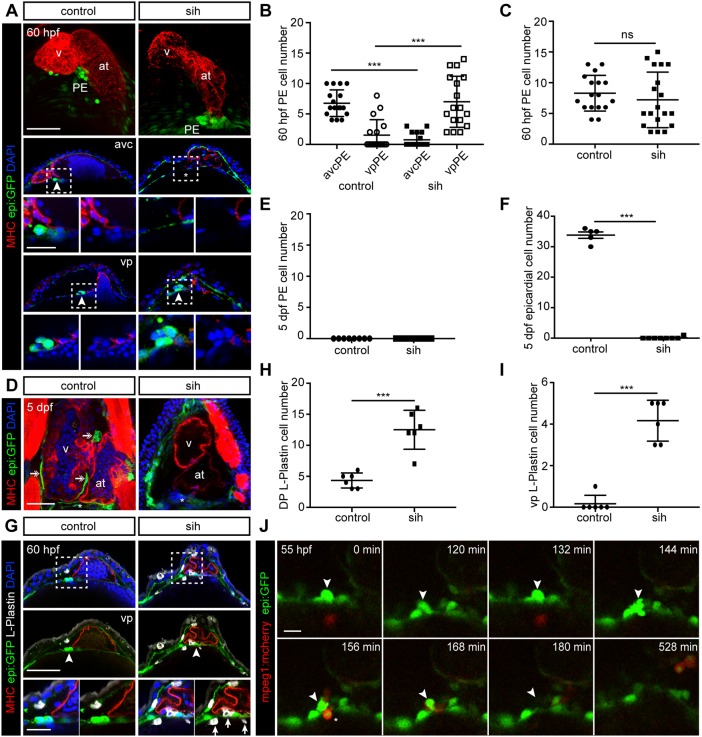
**Heartbeat-induced pericardial fluid advections influence proepicardium localization and survival.** (A,D,G) *epi:GFP* animals immunostained for GFP (green), Myosin heavy chain (MHC) (red) and nuclei counterstained with DAPI (blue). (A) Top panels show a 3D projection of 60 hpf zebrafish hearts. Optical sections of AVC and VP regions in middle panels. Zoomed images (bottom panels) are views of the proepicardium (PE) region. Control siblings were compared with *sih* embryos. Arrowheads, PE cells. Asterisk indicates expected avcPE location. (B) Quantification of avcPE and vpPE cell numbers in A. (C) Quantification of total PE cell number in A. (A-C) Data from ≥4 biological and ≥4 technical replicates. (D) Optical sections of control or *sih* embryos at 5 dpf. Arrows, epicardial cells. Asterisks mark the region where the PE was at 60 hpf. (E) Quantification of total PE cell number at 5 dpf. (F) Quantification of epicardial cell number at 5 dpf. E,F, data from ≥4 biological and two technical replicates. (G) Optical sections at 60 hpf of *epi:GFP* or *sih* embryos immunostained for L-plastin (white). Zoomed images (bottom panels) are views of the PE region. Arrowheads, PE cells; arrows, L-plastin^+^ leukocytes. (H,I) Quantification of L-plastin^+^ cells in the dorsal pericardium (DP) (H), and close to the VP (I) at 60 hpf. Six biological replicates from one experiment. (J) Frames of an *epi:GFP; mpeg1:mCherry* embryo *in vivo* time-lapse starting at 55 hpf (see also Movie 5). Mpeg1-mCherry (red) signal shows macrophages. One optical section after drift correction is shown. Representative of ≥4 biological from ≥2 technical replicates. Arrowheads, emerging PE cell; asterisks, leukocytes. at, atrium; avc, atrio-ventricular canal; dpf, days post-fertilization; hpf, hours post-fertilization; sih, *silent heart*; v, ventricle; vp, venous pole. DP digitally isolated in 3D projections. Representative images from ≥3 biological and ≥2 technical replicates. Scale bars: A,D,G, 50 µm; zoomed images 20 µm; J, 10 µm. Data are mean±s.d., two-tailed Student's *t*-test. ns, not significant; ***P<0.001.

PE cells emerge to a small extent from the DP close to the VP of the heart (vpPE) and to a larger extent from the DP close to the AVC (avcPE) (Peralta et al., 2013). It was previously shown that heartbeat-evoked pericardial fluid forces are essential for PE cell detachment and transfer to the heart (Peralta et al., 2013; Plavicki et al., 2014). It remained unknown whether the heartbeat was also essential for PE formation. We therefore assessed PE formation in the *troponin-2* (*tnnt2*) null mutant line *silent heart* (*sih*) (Sehnert et al., 2002), in which the heartbeat is completely blocked. Similar to control wild-type siblings, *sih* mutants formed PE clusters at 60 hpf (Fig. 5A). However, PE cells were mispositioned and close to the VP in *sih* mutants (Fig. 5A-C). At 5 days post-fertilization (dpf) in *sih* animals, PE cells were no longer present, and no epicardial layer was formed (Fig. 5D-F). We hypothesized that PE cells in *sih* mutants disappear through phagocytosis by macrophages. To test this, we first performed immunostaining against the pan-leukocyte marker L-plastin and found increased number of leukocytes in the pericardial cavity of *sih* mutants (Fig. 5G,H). We found significantly higher numbers of L-plastin^+^ cells close to the VP of the heart in *sih* versus control embryos (Fig. 5I). To further investigate the activity of macrophages, we crossed *epi:GFP* into the macrophage reporter line *mpeg1:mCherry* (Ellett et al., 2011) and injected embryos with a *tnnt2* morpholino. Live imaging of the cardiac region from 55 hpf onwards revealed phagocytosis of PE cells (Fig. 5J; Movie 5). We conclude that PE cells that fail to release into the pericardial cavity and attach to the myocardium in the absence of cardiac contraction are eliminated through phagocytosis. Overall, these results reflect the importance of pericardial fluid advections in PE positioning and supporting PE cell viability.

### Proepicardium formation depends on actomyosin dynamics

**Fig. 6.**
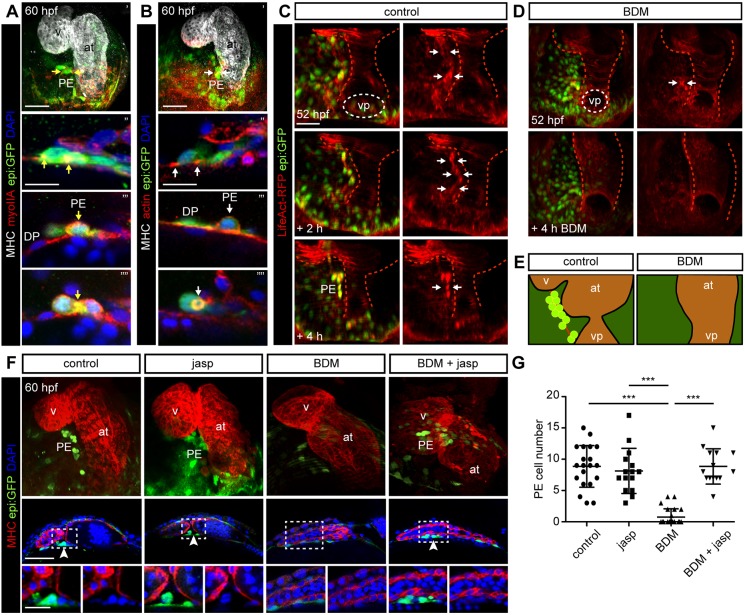
**Proepicardium formation depends on actomyosin dynamics.** (A,B,F) 60 hpf *epi:GFP* animals immunostained for GFP (green) and nuclei counterstained with DAPI (blue). (A) Myosin heavy chain (MHC) (white), Myosin II-A (red). Upper panel, maximum intensity projection of a heart. The three zoomed images are different views of the proepicardium (PE) region. Yellow arrows, Myosin II-A concentration sites. (B) MHC (white), phalloidin-488 to visualize F-actin (red). Upper panel, maximum intensity projection of a heart. The three zoomed images are different views of the PE region. White arrows, actin concentration sites. Representative of ≥3 biological and three technical replicates. (C,D) Ventral view of the heart region in *epi:GFP; LifeAct-RFP* embryos, untreated (C) or treated with 2,3-butanedione monoxime (BDM) (D), shown at different time points from an *in vivo* time lapse. The DP was digitally isolated. The heart tube shape was drawn with a red dashed line. White arrows, sites of actin concentration in DP cells. ≥3 biological replicates from ≥2 experiments. (E) Scheme of actin concentration (red) at DP and PE clusters (green) during PE formation in untreated or BDM-treated embryos. (F) Top panels, 3D projection of a 60 hpf heart (MHC, red). Middle panels, optical section. Bottom panels, zoomed images. Embryos untreated or treated with combinations of jasplakinolide (jasp) and BDM. White arrowheads, PE cells. Representative data from ≥4 biological and three technical replicates. (G) Quantification of PE cell number in F. Animals treated with jasp or BDM+jasp had 8±4 and 9±3 PE cells, respectively (*n*=15 or *n*=14 embryos each), which resembled control conditions, whereas BDM-treated animals presented 1±1 PE cells (*n*=13). at, atrium; DP, dorsal pericardium; h, hours; hpf, hours post-fertilization; v, ventricle; vp; venous pole. Representative images from ≥3 biological and≥2 technical replicates. Scale bar: 50 µm; zoomed images 20 µm; 10 µm in A″-A″″,B″-B″″. Data are means±s.d. Kruskal–Wallis test. ***P<0.001.

Cytoskeleton reorganization is fundamental for the apical extrusion of apoptotic epithelial cells and for cell migration and/or invasion of metastatic cells (Kadeer et al., 2017; Saitoh et al., 2017; Wu et al., 2015). Prior to extrusion, cells present an overall increase in F-actin levels and experience changes in actomyosin localization, from basal to apico-cortical deposition (Martin and Goldstein, 2014). The Myosin II inhibitory drug 2,3-butanedione monoxime (BDM) interferes with Myosin-ADP-P_i_ phosphate release, and locks Myosin II into a low-affinity conformation with actin that impedes Myosin movement on actin filaments. BDM treatment reversibly stops the heart and impairs PE formation (Peralta et al., 2013). Since our results exclude a role for the heartbeat in PE extrusion, the action of BDM on PE formation might not be through the inhibition of cardiac contraction. Alternatively, BDM might interfere with Myosin II function within PE precursor cells. We therefore decided to have a closer look at actomyosin structures and reorganization during PE formation. Immunofluorescence analysis revealed that Myosin II-A was highly expressed in PE cells (Fig. 6A′). In DP cells close to the midline, Myosin II-A signal was strong at the cell boundaries (Fig. 6A″). In PE cells, Myosin II-A was located cortically and at boundaries between PE cell pairs (Fig. 6A‴,A″″). *In vivo* imaging of the *actb2:myl12.1-mCherry*^e1954^ line, expressing Myosin II-mCherry under the promoter *actin beta 2* (*actb2*), confirmed the accumulation of Myosin II in DP cells at the midline and in the PE cluster (Movie 6). We also analyzed the localization of polymerized actin at 60 hpf using a phalloidin-coupled fluorophore (Fig. 6B′). Whereas actin was located in the basal region of DP cells (Fig. 6B″,B‴), it was polarized apico-cortically in PE cells, consistent with the pattern observed for Myosin II (Fig. 6B‴,B″″). *In vivo* characterization of actomyosin dynamics in *LifeAct-RFP*; *epi:GFP* embryos showed that from 52-60 hpf F-actin concentrated in DP cells at the midline where the PE appears (Fig. 6C). Indeed, a thick actin cable was visible in *epi:GFP*^+^ cells at the time of PE formation, spanning the midline of the DP (Movies 7-9). Actin was concentrated basally in DP cells, whereas in PE cells F-actin became localized cortically and concentrated in the contact region between the rounded up PE cells (Fig. 6B‴; Movie 10). Temporally associated with PE cell extrusion, we observed actin-ring-like structures beneath the emerging cell (Movie 11). Similar forming and contracting actin rings were recently described during apically extruding peridermal cells (Schepis et al., 2018). In a final stage prior to release, a thin actin-rich stalk was still visible between PE cells and underlying DP cells (Movie 12). In sum, our data suggest that PE formation occurs concomitantly with extensive actin reorganization: before PE formation, F-actin is located basally in DP cells, then it becomes concentrated in PE precursor cells and it finally accumulates all around the cortex of the rounded PE cells that ultimately become apically extruded.

We next investigated how BDM affects actomyosin dynamics during PE formation. BDM treatment led to a decrease in LifeAct-RFP signal in the DP at the midline (Fig. 6D; Movie 13), suggesting that F-actin is unstable in DP cells when Myosin II is inhibited (Fig. 6E). We reasoned that if actin polymerization was required for PE formation, pharmacological enhancement of actin stability should counteract the effect of Myosin II inhibition. To test this hypothesis, we administered jasplakinolide (jasp), which promotes actin filament polymerization and stabilization, to *epi:GFP* animals in the presence or absence of BDM. PE cell number in animals treated with jasp or BDM+jasp resembled control conditions, whereas BDM-treated animals presented fewer PE cells (Fig. 6F,G). Thus, enhancement of actin filament polymerization and stabilization in the presence of BDM correlated with PE formation, suggesting that a stable F-actin network is necessary for PE formation.

### Bmp signaling positively regulates actomyosin dynamics during proepicardium formation

**Fig. 7.**
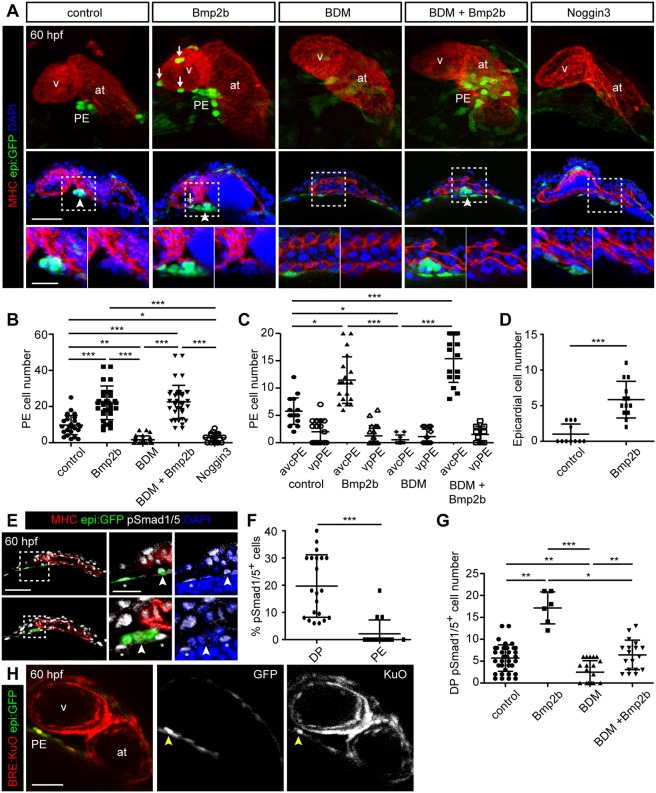
**Bmp2b rescues proepicardium formation upon Myosin II inhibition.** (A,E) *epi:GFP* embryos immunostained for GFP (green), Myosin heavy chain (MHC) (red) and nuclei counterstained with DAPI (blue). (A) Top panels, 3D projection of a 60 hpf zebrafish heart, middle panels optical section and zoomed images below. Control compared with embryos overexpressing *bmp2b* with and without 2,3-butanedione monoxime (BDM) or with those overexpressing *Noggin 3*. Arrowheads, PE cells; arrows, epicardial cells. (B,C) Quantification of PE cell number (B) and avcPE and vpPE cell number (C) in conditions shown in A. (D) Quantification of epicardial cell number at 60 hpf after *bmp2b*-overexpression versus non-overexpression embryos. (E) Optical sections at 60 hpf showing anti-pSmad1/5 staining. Zoomed views below. Arrowheads, PE cells; asterisks, DP cells. (F) Percentage of pSmad1/5^+^ cells in the DP compared with the PE in control animals. (G) Total number of pSmad1/5^+^ cells in the DP at 60 hpf. (H) Optical section from an *in vivo* time lapse of a Bmp reporter line, *BRE*:*KuO*; *epi:GFP* embryo. Yellow arrowheads, double-positive PE cells. at, atrium; DP, dorsal pericardium; hpf, hours post-fertilization; PE, proepicardium; v, ventricle. DP digitally isolated in 3D projections. Representative images from ≥3 biological and ≥2 technical replicates. Scale bars: 50 µm; 20 µm in zoomed A,E. Data are mean±s.d., Kruskal–Wallis test (B,C,G), two-tailed Student's *t*-test (D,F). *P<0.05; **P<0.01, ***P<0.001.

We next sought to determine whether the developing myocardium, and signals such as Bmp derived from it, influence PE formation through the regulation of actomyosin dynamics in the DP. While it is known that Bmp regulates PE formation (Liu and Stainier, 2010), the specific time point of action has not been well described. Therefore, we further dissected the developmental time point when Bmp signaling acts on PE formation. We performed experiments using the transgenic line *hsp70:bmp2b* (Chocron et al., 2007) crossed into *epi:GFP*, which allows the increase of *bmp2b* levels at a specific developmental stage by heat shocking (HS) the embryos. In control (non-transgenic for *hsp70:bmp2b*) animals subjected to HS, the PE was completely formed and visible at 60 hpf and comprised ∼10 cells (Fig. 7A,B). However, overexpression of *bmp2b* during the embryonic stages preceding PE formation resulted in a twofold increase of PE cell number (Fig. 7A,B). To assess whether Bmp2b acts on a particular subpopulation, we quantified the number of avcPE and vpPE cells and found that *bmp2b* overexpression significantly increased the number of cells in the avcPE cluster (Fig. 7C). Consistent with this finding, antagonizing Bmp signaling by overexpressing *noggin3* in the *hsp70l:noggin3^fr14^* (Chocron et al., 2007) line through HS at 48 hpf significantly decreased the number of PE cells (Fig. 7A,B). Additionally, there were more epicardial cells on the ventricular myocardial surface in *bmp2b*-overexpressing fish at 60 hpf (Fig. 7A,D). We explored whether the enlarged PE clusters observed upon *bmp2b* overexpression were a consequence of an expanded population of DP cell precursors. However, the total number of DP cells at 48 hpf prior to PE formation did not differ between *bmp2b*-overexpressing fish and control animals heat shocked at 26 and 32 hpf (Fig. S3A; *n*=6/7 animals), ruling out this possibility. Thus, *bmp2b* acts on PE formation during the time window of extrusion, but does not regulate the number of early progenitor cell populations.

We next aimed to assess whether ectopic Bmp2b activated Bmp signaling directly in PE cells. To do this, we evaluated the expression of its downstream effector pSmad1/5 (Fig. 7E-G). Approximately 20% of DP cells were pSmad1/5^+^, whereas a mean of only 2% of PE cells were pSmad1/5^+^ (0±1 positive cells from a total of 9±4 cells; *n*=16 embryos). The percentage of pSmad1/5^+^ DP cells was significantly higher than the percentage of pSmad1/5^+^ PE cells (*P*<0.001). Moreover, the total number of pSmad1/5^+^ DP cells was increased in *bmp2b*-overexpressing embryos (Fig. 7G). We also used a Bmp reporter line expressing Kusabira Orange (KuO) controlled by a promoter harboring several Smad binding sites, named Bmp responsive elements (BRE) (Collery and Link, 2011). *In vivo* imaging of *BRE:KuO*; *epi:GFP* fish revealed that DP cells and some PE cells were KuO^+^ (*n*=1-2 KuO^+^ PE cells per animal, three embryos from 52-58 hpf), confirming that Bmp signaling was transiently active in PE cells (Fig. 7H; Movie 14). Therefore, an increase in Bmp activity within the DP correlates with PE formation.

We aimed to determine whether Bmp signaling promotes PE formation through the regulation of actomyosin dynamics. Thus, we tested whether overexpression of *bmp2b* rescues impaired PE cluster formation upon BDM treatment from 48 hpf onwards. PE formation was observed in *bmp2b*-overexpressing animals treated with BDM, and clusters were larger than in controls (23±3 vs 10±5 cells, *P*<0.0001, *n*=29 embryos) (Fig. 7A,B, see also Fig. 7C). Inhibition of PE formation using Myosin II inhibitors (BDM and blebbistatin) was dose-dependent (Fig. S3B), but *bmp2b* overexpression rescued PE formation in BDM- and blebbistatin-treated animals (Fig. S3C,D). We next tested whether the rescue of PE formation was dependent on *bmp2b* expression levels. Larger PE clusters were detected after three HS pulses (at 26, 32 and 48 hpf) (22±9 cells; *n*=25 embryos) (Fig. 7B) than with only one HS pulse at 48 hpf (10±4 cells; *n*=30 embryos) (Fig. S3E). We also assessed at which developmental stage the effect of Bmp2b on PE formation was more prominent. A unique HS at 48 hpf rescued PE formation at 60 hpf in BDM-treated hearts (8±4 vs 3±2 cells, *n*=20 embryos) (Fig. S3E), but a single HS at 26 hpf did not rescue PE formation at 60 hpf in BDM-treated embryos (6±3 vs 4±2 cells, *n*=12 embryos) (Fig. S3F). *Bmp2b* overexpression after BDM treatment also failed to rescue PE formation (Fig. S3G, *n*=10).

**Fig. 8.**
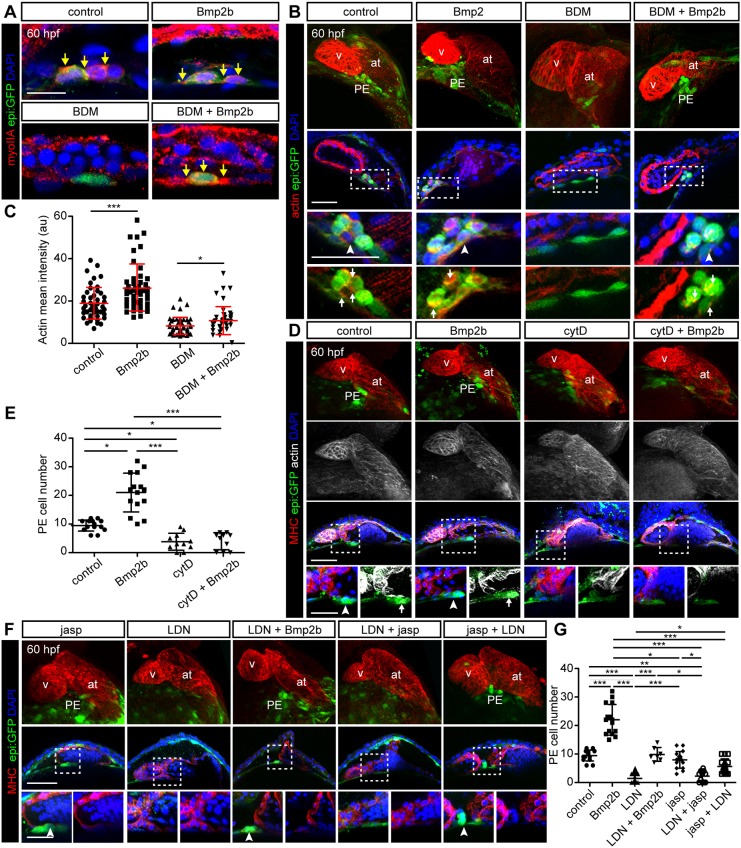
**Bmp2b controls actin rearrangements necessary for proepicardium formation.** (A,B,D,F) *epi:GFP* embryos at 60 hpf immunostained for GFP to detect dorsal pericardial (DP) and PE cells (green) and DAPI-counterstained nuclei (blue). (A) Anti-myosin II-A immunostaining (red). Zoomed view of the PE area on untreated fish compared with those overexpressing *bmp2b* with and without 2,3-butanedione monoxime (BDM). Yellow arrows, Myosin II-A apico-lateral accumulation in PE cells. (B) F-actin is detected with fluorescently labeled phalloidin (red). Top panels, maximum intensity projection of embryo hearts. Middle panels, optical sections. Bottom panels, zoomed images of PE region. Untreated fish were compared with those overexpressing *bmp2b* with and without BDM. Arrowheads, PE cluster; arrows, actin concentration sites in the PE cluster. (C) Quantification of actin intensity (arbitrary units) in PE cells from conditions shown in B. (D) Top images, 3D projections of hearts from control or *bmp2b*-overexpressing fish treated with cytochalsin D (cytD). Immunostained for MHC (red) and phalloidin to detect actin (white). Middle panels, optical sections of the hearts. Bottom panels, zoomed images of PE region. Arrowheads, PE cluster; arrows, F-actin concentration sites in the PE. (E) Quantification of PE cell number as shown in D. (F) Hearts immunostained for MHC (red) from experimental conditions as indicated. Top row, 3D projection; middle row, optical sections; bottom row, zoomed views of the PE region. Arrowheads, PE cells. (G) Quantification of PE cell number from conditions shown in F. Data are mean±s.d., two-tailed Student's *t*-test (C), Kruskal–Wallis test (E,G). *P<0.05, **P<0.01, ***P<0.001. at, atrium; hpf, hours post-fertilization; PE, proepicardium; v, ventricle. DP digitally isolated in 3D projections. Representative images from ≥3 biological and ≥2 technical replicates. Scale bars: 10 µm in A; overview 50 µm and zoomed views 20 µm in B,E,F.

To understand the mechanisms through which Bmp2b restores PE formation in the presence of BDM, we explored how the actomyosin network is altered by *bmp2b* overexpression. BDM treatment reduced the amount of Myosin II-A in DP cells (*n*=8 animals), and overexpression of *bmp2b* in BDM-treated animals rescued the apical polarization of Myosin II-A in PE cells (*n*=11) to an extent similar to that observed in control embryos (*n*=10) (Fig. 8A). These results suggest a recovery of actomyosin dynamics by the Bmp pathway. Thus, we investigated how actin polymerization is affected upon *bmp2b* overexpression. Examination of F-actin at 60 hpf revealed that *bmp2b* overexpression significantly increased F-actin levels. In the presence of BDM, actin levels in PE cells were lower than in controls; however, this was significantly rescued upon *bmp2b* overexpression (Fig. 8B,C).

In agreement with a role of F-actin on PE formation, treatment with the actin polymerization and elongation inhibitor, cytochalasin D at 2 µM from 48 hpf onwards, significantly decreased the PE size (*n*=14 or 11 embryos per group) at 60 hpf. Of note, *bmp2b* overexpression was not sufficient to rescue PE formation in the presence of cytochalasin D (Fig. 8D,E). This further confirms that Bmp can only induce PE formation in the presence of an intact F-actin network.

We next tested whether the effect of Bmp inhibition on PE formation could be rescued by increasing actin stabilization. The number of cells in PE clusters of animals treated with the Bmp receptor-I inhibitor LDN-193189 (LDN) (Björklund et al., 2008; Cuny et al., 2008) was compared with that of animals treated with a combination of LDN and jasp. LDN treatment reduced the number of PE cells per cluster compared with untreated controls. It also counteracted the effect of *bmp2b* overexpression on PE size (Fig. 8F,G). In the LDN-treated group (*n*=24) as well as LDN+jasp groups (*n*=9), the mean number of PE cells was reduced by 20-30% compared with untreated controls. By contrast, when we stabilized actin filaments with jasp from 48 hpf onwards and 4 h prior to LDN administration, the number of PE cells was significantly increased to levels comparable with controls (*n*=11). Overall, these results suggest that Bmp signaling influences actomyosin polymerization, and the stabilization of the F-actin network partially compensates for the negative effect of Bmp signaling inhibition on PE formation.

### Bmp2 overexpression rescues dorsal pericardial cell displacement towards the midline upon Myosin II inhibition

**Fig. 9.**
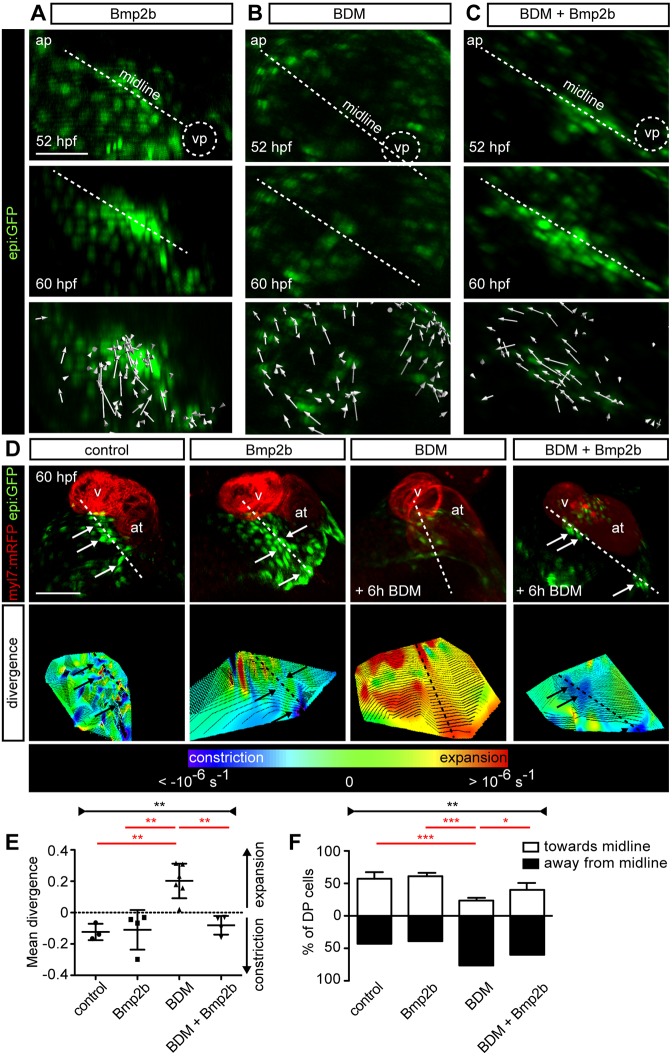
**Effect of Bmp2b under Myosin II inhibition on dorsal pericardial layer constriction.** (A-C) First and last frame of an *epi:GFP in vivo* time lapse of *bmp2b*-overexpressing (A), 2-3-butanedione monoxime (BDM)-treated (B) or *bmp2b* overexpression in BDM-treated larvae (C); midline is shown by a dashed white line. Arrows indicate overall direction of tracked cells. (D) Ventral view of *epi:GFP; myl7:mRFP* embryo. Shown are 3D projections of untreated, *bmp2b*-overexpressing, BDM-treated, and *bmp2b*-overexpression in BDM-treated fish. Bottom images, 3D projection of the divergence field of the DP at 60 hpf; blue regions represent tissue constriction and red-orange regions represent tissue expansion. Arrows, proepicardial cells; dashed lines mark the midline. (E) Mean divergence of tracked cells within the DP. (F) Percentage of cells moving towards or away from the midline. Data are mean±s.d., Kruskal–Wallis test (black labels); two-tailed Student's *t*-test (red labels).*P<0.05, **P<0.01, ***P<0.001. ap, arterial pole; at, atrium; hpf, hours post-fertilization; v, ventricle; vp, venous pole. Dorsal pericardium (DP) digitally isolated in 3D projections. Representative images from ≥3 biological and ≥2 technical replicates. Scale bars: 50 µm.

To gain deeper insight into the mechanisms of Bmp2b action, we analyzed how DP cell displacement is altered in BDM-treated animals in a background of *bmp2b* overexpression. We imaged *epi:GFP* animals from 52-60 hpf and tracked *epi:GFP*^+^ cells in the DP. In *bmp2b*-overexpressing animals, the DP constricts to the midline (Fig. 9A), as observed in controls (Fig. 1C). Upon BDM treatment, the typically observed crowding of GFP^+^ cells at the midline was not apparent (Fig. 9B). However, *bmp2b* overexpression rescued DP cell displacement towards the midline upon BDM treatment (Fig. 9C). We next quantified the DP cell divergence. BDM treatment led to an overall DP tissue expansion, whereas *bmp2b* overexpression led to DP tissue constriction, similar to control embryos or the control condition (Fig. 9D,E). *bmp2b* overexpression under BDM treatment rescued the constriction of the DP tissue (Fig. 9D,E). Accordingly, cell displacement tracking revealed a movement towards the midline in control (*n*=4) embryos and *bmp2b*-overexpressing (*n*=4) embryos, whereas DP cells were predominantly displaced away from the midline in BDM-treated embryos (*n*=5) (Fig. 9F). Again, *bmp2b* overexpression in BDM-treated animals favored the movement towards the midline (*n*=3). Taken together, these results suggest that Bmp signaling modulates actomyosin contractility to allow the displacement of DP cells towards the midline, which ultimately leads to PE formation.

## DISCUSSION

We describe a mechanism by which viable cells are extruded apically from a mesothelial tissue to fulfill a developmental fate (Fig. S4). Apical extrusion controls epithelial layer homeostasis in the intestine while preserving its barrier function (Knodler et al., 2010; Ritchie et al., 2012; Simovitch et al., 2010). Live cell extrusion also occurs in embryonic epithelia to control cell number (Eisenhoffer et al., 2012). An increase in cellular density leads to the elimination of supernumerary cells towards the apical side (Eisenhoffer et al., 2012; Marinari et al., 2012) and extruded cells undergo apoptosis upon loss of cell contact with their neighbors (Gibson and Perrimon, 2005; Rosenblatt et al., 2001). Normal epithelial cells also play an active role in this process by accumulating actin cytoskeleton and intermediate filament proteins at the interface with adjacent transformed cells (Kajita et al., 2014). Instances where extruded cells survive have been mostly documented for transformed cells (Hogan et al., 2009; Kajita et al., 2010; Leung and Brugge, 2012; Vidal et al., 2006). Here, we describe a physiological mechanism of apical cell extrusion that results in cell survival, and is part of a natural process occurring during heart development that is required for the generation of epicardial precursor cells. The mechanism of cell extrusion during the emergence of hematopoietic stem cells from the dorsal aorta might be similar to PE cell extrusion (Bertrand et al., 2010; Kissa and Herbomel, 2010; Lancino et al., 2018; Zovein et al., 2008). The existence of such processes in different developing tissues might indicate a more conserved role of extrusion mechanisms in organ formation, and these are just the first examples of cell extrusion to generate progenitor cells with a developmental fate.

The gene regulatory mechanisms that promote the survival of extruded PE cells, until they attach to the myocardium, remains unknown. We show that in the absence of a heartbeat, macrophages remove extruding cells that are not released into the pericardial cavity owing to the absence of pericardial fluid flow. It is plausible that the rapid attachment of extruded PE cells to the myocardial surface promotes their survival.

The interplay between cell signaling and mechanics during development is a major focus of research (Miller and Davidson, 2013). An outstanding question to resolve is how Bmp2 signaling and actomyosin dynamics are linked to control PE formation. Our results reveal a role for pericardial cell movements in PE formation. These motions are dependent on the actomyosin cytoskeleton and lead to an overall constriction of the DP tissue at the midline. Interestingly, actomyosin-dependent cell rearrangements in the DP also influence outflow tract development in the mouse (Francou et al., 2017). We propose a model in which Bmp promotes actin polymerization, dynamics and/or stability, leading to PE formation (Fig. S4). Bmp2b might counteract the tissue compliance caused by inhibition of the actomyosin network. Bmp2b might also stabilize F-actin; since actin concentration was higher upon *bmp2b* overexpression, an even higher dose of BDM would be required to disrupt the actomyosin network. This would also explain why jasp treatment rescues PE formation upon BDM administration. Alternatively, Bmp2 might rescue the F-actin tension by modulating the expression of other non-conventional myosins not inhibited by BDM. Indeed, Bmp signaling regulates epithelial morphogenesis by upregulating Myh9 (Zhao et al., 2015), and controlling F-actin rearrangements (Jidigam et al., 2015). Along this line, during retinal morphogenesis, actomyosin forces that are blocked when Myosin II is inhibited with BDM can be rescued by expression of BDM-insensitive myosins (Norden et al., 2009). Furthermore, Bmp2 controls the expression of the *non-muscle myosin Va*, promoting cellular migration (Zhang et al., 2014). Bmp2 signaling also regulates *Prrx1* expression in lateral plate mesoderm, which in turn regulates the expression of Palladin, an actin-bundling protein (Ocaña et al., 2017). These signaling cascades, in which Bmp controls actomyosin dynamics, might be also important for PE formation.

In conclusion, our findings illustrate the importance of an intact actin scaffold for generating the interactions and forces between DP cells necessary for the extrusion of PE cells, the source of the epicardial layer. Collectively, our results suggest an orchestration between heart tube maturation and PE formation, and may represent a paradigm for the coordinated action of signaling molecules and mechanical forces in controlling tissue morphogenesis.

## MATERIALS AND METHODS

### Zebrafish strains and husbandry

All animal experiments were approved by the Community of Madrid ‘Dirección General de Medio Ambiente’, Spain, and the ‘Amt für Landwirtschaft und Natur’ from the Canton of Bern, Switzerland. Animals were housed and experiments performed in accordance with Spanish and Swiss bioethical regulations for the use of laboratory animals. Fish were maintained at a water temperature of 28°C. The following fish were used: wild-type AB strain, *Et*(*-26.5Hsa.WT1-gata2:EGFP*)^cn1^ (*epi:GFP*) (Peralta et al., 2013), *Tg*(*myl7:mRFP*) (Rohr et al., 2008), *Tg*(*hsp70l:bmp2b*)^fr13^ and *Tg*(*hsp70l::noggin3*)^fr14^ (Chocron et al., 2007), *Tg*(*βactin:LifeAct-RFP:RFP*)^e2212Tg^ (Maitre et al., 2012), *Tg*(*actb2:myl12.1-mCherry*)^e1954^ (Behrndt et al., 2012), *Tg*(*BRE-AAVmlp:dmKO2*)^mw40^ (Collery and Link, 2011), *tnnt2a*^b109/b109^ (Sehnert et al., 2002) and *Tg*(*mpeg1:mCherry*) (Sanz-Mórejon, Garcia-Redondo et al., unpublished).

The *Et*(*-26.5Hsa.WT1-gata2:EGFP*)^cn1^ line contains a reporter construct flanked by FRT sites, in which cardiac actin drives the expression of RFP. The cassette was removed by injection of flipase into one-cell stage embryos. We named this new line *Et*(*-26.5Hsa.WT1-gata2:EGFP*)^cn14^.

### Heat shock

Heat shock was performed on the embryos at 39°C in preheated water for 1 h. Animals treated with heat shock were genotyped after analysis. This allowed unbiased comparison and blinded quantification of experimental and control groups.

### *Par3-RFP* mRNA synthesis and microinjection

The Par3-RFP plasmid was kindly provided by the group of Jon Clarke, King's College, London, UK (Alexandre et al., 2010). The plasmid was transcribed by using the standard protocol and compounds from the mMESSAGE mMACHINE SP6 kit (Ambion) and purified with the RNA Clean & Concentrator-5 kit (Zymo Research). Microinjection of 150 ng/µl purified mRNA was performed at the one-cell stage.

### Immunofluorescence

Embryos were fixed overnight in 4% paraformaldehyde in PBS, washed in 0.01% PBS-Tween-20 (Sigma-Aldrich) and permeabilized with 0.5% Triton X-100 (Sigma-Aldrich) in PBS for 20 min. Several washing steps were followed by blocking for 2 h with 5% goat serum, 5% BSA, 20 mM MgCl_2_ in PBS followed by overnight incubation with the primary antibody at 4°C. Secondary antibodies were diluted in PBS and incubated for 3 h. Nuclei were counterstained with DAPI (Invitrogen) for 30 min. After several washes, embryos were mounted in Vectashield (Vector). Immunofluorescence staining on paraffin sections was performed as described in Gonzalez-Rosa et al. (2011).

The antibodies and stains for immunofluorescence detection were as follows: anti-myosin heavy chain (MF20, ab_2147781, DSHB) at a 1:20 dilution, anti-pH3 (06-570, Merck) at 1:100, anti-GFP (1010, Aveslab) at 1:1000, anti-pSmad1/5 (9516, Cell Signaling Technology) at 1:100, phalloidin-488 (A12379, Thermo Fisher) at 1:100, anti-β-catenin (610153, BD Transduction Laboratories) at 1:200, anti-laminin (L9393, Sigma-Aldrich), anti-myosin II-A (M8064, Sigma-Aldrich) at 1:100 and anti L-plastin (kind gift of Paul Martin, University of Bristol, UK) at 1:100. Secondary antibodies were the following: Biotin-SP goat anti-rabbit IgG (111-066-003, Jackson ImmunoResearch), streptavidin Cy5 (SA1011, Invitrogen), anti-mouse IgG2b Alexa Fluor 568 (A21144, Thermo Scientific), anti-chicken Alexa Fluor 488 (A11039, Thermo Scientific) and goat anti-rabbit IgG Alexa Fluor 568 (A11036, Thermo Scientific), all diluted at 1:500.

Embryos were imaged with a Zeiss 780 confocal microscope fitted with a 20× objective 1.0 NA with a dipping lens. *Z*-stacks were taken every 3-5 µm. Maximal projections of images were 3D reconstructed in whole-mount views using Imaris software (Bitplane). The pericardial ventral tissue was digitally removed to provide a clearer view of the heart. Optical sections of 1-3 *z*-slices were also reconstructed.

### Quantification of DP and PE cells

PE cells have been described to emerge from two main regions of the DP: the avcPE appears close to the AVC, and the vpPE around the VP. We counted each cell in each *z*-plane by DAPI nuclear counterstain and GFP expression using the line *epi:GFP*. We took care not to count any cell twice. Cells with a round morphology at the VP or AVC region were counted as PE cells, while cells with a flat morphology in the DP were counted as DP cells. See Fig. S5 and Movie 15 for further explanation.

### Pharmacological treatments

Embryos were manually dechorionated and incubated with compounds from 48 hpf onwards (unless otherwise stated). The following compounds were used: aphidilcolin (150 µM), hydroxyurea (20 µM), BDM (10-20 mM), LDN-193189 (20 mM), cytocalasin-D (2 µM) (all from Sigma-Aldrich), blebbistatin (25-50 µM; Abcam) or jasplakinolide (0.15 µM; Thermo Fisher).

### *In vivo* imaging

Embryos were transferred to fish water containing 0.2 mg/ml tricaine (Sigma) and 0.0033% 1-phenyl-2-thiourea (Sigma-Aldrich), and immobilized in 0.7% agarose (NuSieve GT Agarose, Lonza) in a 35-mm Petri dish with a glass cover (MatTek Corporation). Zebrafish hearts were scanned bidirectionally at 30 frames per second (fps) with an SP5 confocal microscope (Leica) using a 20× glycerol immersion objective with 0.7 NA. Videos were acquired every 5 µm, with a line average of 6 and a pinhole of 1.9 AU. Around 65 *z*-stack videos were acquired per heart every 10-15 min. GFP, red and brightfield channels were acquired simultaneously.

High-resolution *in vivo* imaging was performed with the Zeiss LSM880 airy scan fastmode, using a 40×/1.1 water immersion objective lens. Sampling was performed with 1× Nyquist coefficient parameters. Airy scan processing was performed in ZEN 2.3 (Zeiss).

Realigned 4D data sets were displayed and analyzed using Imaris or ImageJ.

### Deconvolution

Deconvolution was performed with the Huygens Remote Manager (SVI). The theoretical point spread function needed for deconvolution was calculated from the acquisition parameters and the microscope specifications. The image with increased resolution and enhanced signal-to-noise ratio is represented.

### Digital isolation of the dorsal pericardium

In *epi:GFP* embryos all pericardial cells express GFP. To visualize and track DP cells in 3D the VP was removed from all images of a *z*-stack. For single time-point 3D reconstructions the surface function in Imaris was used and a mask was created to isolate the DP digitally. To remove the VP in 3D *in vivo* time-lapse movies, surfaces were created around the DP every 20 frames in Imaris. Binary images of the surfaces were exported to Fiji. A customized macro was applied to interpolate the surfaces over time for every single frame. Subsequently, the interpolated mask was applied to the 3D reconstruction to isolate the DP in all frames.

### Drift correction

Small movements of the plate and the embryo in the agarose were manually suppressed using a drift correction, selecting a particle, usually a noise voxel, following the movement to be corrected in two consecutive frames. Moreover, a posterior drift correction was applied to suppress the intrinsic movement of the developing embryo. Microscope and growth drift was corrected in Imaris. The VP was identified as a stable reference structure and tracked in each frame. Subsequently, the ‘Correct drift’ tool was applied to the resulting track.

### Cell tracking

Cell tracking was performed using a built-in Imaris tool, allowing for an automatic creation of 4D trajectories of dorsal pericardial cells. A region of interest was manually selected and, after applying a background subtraction algorithm ‘Gaussian filter’, an autoregressive algorithm was applied to perform cell tracking. Such an autoregressive algorithm works under the assumption that particles move from frame to frame in a quasi-predictive fashion, interpolating the data from previous frames. This approach is pertinent in cells embedded in a tissue and hence was chosen. 4D trajectories were further filtered in terms of duration and overall displacement to remove false-positive trajectories that would add noise to further calculations.

### Divergence of velocity field calculator tool

The algorithm to obtain the divergence of the dorsal pericardium velocity field (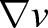) was determined as follows: divergence of the velocity field (∂*v*) associated with the movements of the dorsal pericardium in the *x*, *y* and *z* planes was calculated as an indicator of the expansion of the tissue. The divergence is a mathematical operator that, when applied to a velocity field, indicates how much a set of particles expand or constrict in space. In this particular case, the set of particles is given by the dorsal pericardial cells, hence giving an estimate of the expansion/constriction of the tissue and characterizing it kinematically. It is mathematically described in Eqn 1,(1)
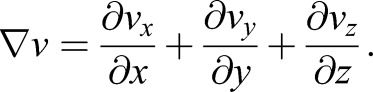
To do so, a customized MATLAB code was created. Taking as an input the 4D trajectories calculated with Imaris, a 2D grid was created, which was used to interpolate the *z*-position of the dorsal pericardial cells, hence resembling the geometry of the dorsal pericardium. The spacing of the grid satisfies the Nyquist criteria, such that Δ*x*_grid_<2×size of the cell nucleus. This interpolation was calculated using a Delaunay triangulation method. In a similar procedure, each velocity component was interpolated across the mesh using a spline method. The interpolated velocity field and *z*-position were used to calculate the divergence of the velocity field. Since the divergence calculated takes into account three dimensions, but the geometry of the tissue is a laminar 2D surface embedded in a 3D space, 2D divergences were taken in the *xy*, *yz*, *xz* planes and algebraically operated to obtain the 3D divergence as described in Eqn 2,(2)
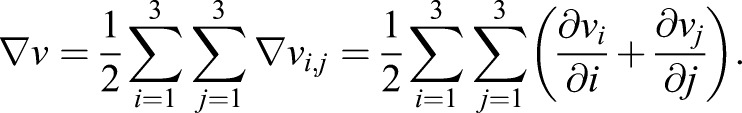
After calculation of the divergence field each node of the *z*-interpolated surface is assigned a divergence value, with positive values indicating an expansion and negative values a constriction. Additionally, the code provides the mean divergence of the surface (S) for each time frame, according to Eqn 3,(3)
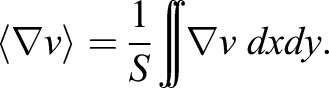
The code will be shared upon request to the authors.

### Angle calculation and directionality to midline

To calculate the angle of cell movement to the midline, *epi:GFP* embryos were *in vivo* imaged from 52 hpf. The midline is a defined line that runs from the VP to the outflow tract of the heart tube. Dorsal pericardium *epi:GFP*^+^ cells were tracked using Imaris software during the time lapse, and the movement vector was calculated regarding the first and the last position of the cell. The angles that form the cell tracks vectors with the midline were calculated in ImageJ. The directionality of the cell tracks in relation to the midline was calculated with regards to the relative distance of the first and last cell track point to the midline vector. Distance of first track point to midline>distance of last track point=cell movement towards midline; distance of first track point to midline<distance of last track point=cell movement against midline.

### Manual interpolation of cell shapes

Image data from *in vivo* movies were visualized in Imaris and a part of the image containing the DP was isolated over time by surface drawing. Images of the isolated DP were exported to Fiji. A line was drawn in parallel to the DP, which was expanded to a rectangle, containing all pixels showing the DP. The re-slice tool was applied to obtain a view of the dorso-ventral plane. The obtained re-slice was subsequently maximum intensity projected. In the first frame, outlines were drawn, describing the LifeAct-RFP signal. LifeAct-RFP and GFP signals were constantly cross-validated, to always have one intensity center of GFP per cell outline. A customized tool for Fiji was used to transfer the shapes from the previous to the subsequent frame. All shapes were manually adapted to the changes in the signal and therefore cell shape changes. Manual interpolation was performed to segment shapes of 47 cells in 60 subsequent frames in each of three datasets. As all objects were maintained over time, a simple indexing system was then used to track changes of individual cells over time. A second customized tool was used to color-code each shape according to its area. The midline was drawn from the VP to the AP, and the relative location of the AVC was chosen as reference point. Data containing information about *xy* location (center of object) and area were exported and analyzed in MATLAB.

### Statistical analysis

Sample size was calculated based on previous experience working on PE formation to ensure adequate power. Embryos that did not develop properly and did not present the morphology expected for their developmental stage were excluded from analysis [except for those overexpressing *bmp2b*, known to present defects in fin development (Peralta et al., 2013)]. Student's unpaired *t*-test for comparisons between two groups or one-way analysis of variance for comparisons between more than two groups was used when normal distribution could be assumed. When the normality assumption could not be verified with a reliable method, the Kruskal–Wallis test was used. Model assumptions of normality and homogeneity were checked with conventional residual plots. The specific test used in each comparison is indicated in the figure legend. Calculations were made with Microsoft Excel and GraphPad Prism. *P*-values are indicated either in the figure legends or the main text or summarized.

## Supplementary Material

Supplementary information
