# Supplementary material for: Actin dynamics and the Bmp pathway drive apical extrusion of proepicardial cells
Source: Development. 2019 Jul 4;146(13):dev174961. doi: 10.1242/dev.174961 (PMC6633599; doi:10.1242/dev.174961)
Supplement: Supplementary information [file develop-146-174961-s1.pdf]

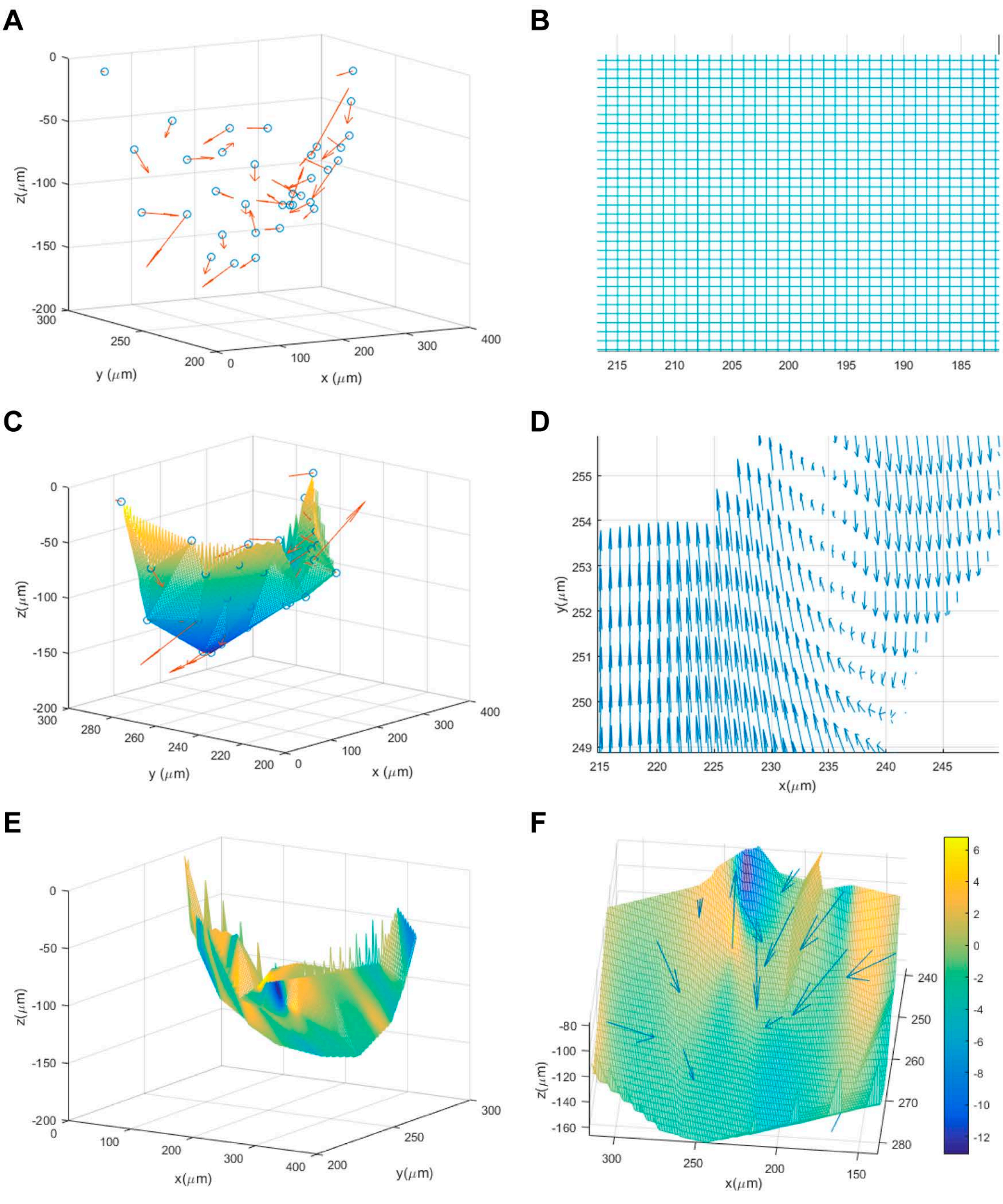

Fig. S1. Divergence of velocity field calculation. (**A**) Blue dots represent the cellular position of dorsal pericardial cells, orange arrows indicate the velocity of each cell at a given time point. Both the position and the velocity are calculated using Imaris (Bitplane) and used as an input for the algorithm. (**B**) 2D grid created for the interpolation of the z-position field to model the geometry of the dorsal pericardium and the velocity field. The spacing of the grid satisfies Nyquist criteria, such that Δxgrid < 2*size of the cell nucleus. (**C**) 3D rendering of the interpolation of the dorsal pericardium z-position. Interpolation was calculated using a Delaunay triangulation method. The color map displays only the z-position. (**D**) 2D section of the interpolated velocity field using the grid in b. The interpolation is used separately for vx, vy and vz, and summed together to obtain the velocity vector v. (**E**) 3D rendering of the interpolated z-position (geometry of the dorsal pericardium) with the color map displaying the divergence of the velocity field. The divergence is calculated as a set of 2D projections in the XY, XZ, YZ planes and computed as the semi-sum of all of them $\nabla \cdot \vec{v} = \frac{1}{2}(\nabla \cdot \overrightarrow{v_{x,y}} + \nabla \cdot \overrightarrow{v_{x,z}} + \nabla \cdot \overrightarrow{v_{y,z}}$. (**F**) Zoom of a section of the interpolated surface coupled with the original velocity vectors (blue arrows). It can be observed that the arrows point inwards –constriction– with the resulting negative values of the divergence of the velocity field (blue in the color map).

Development • Supplementary information

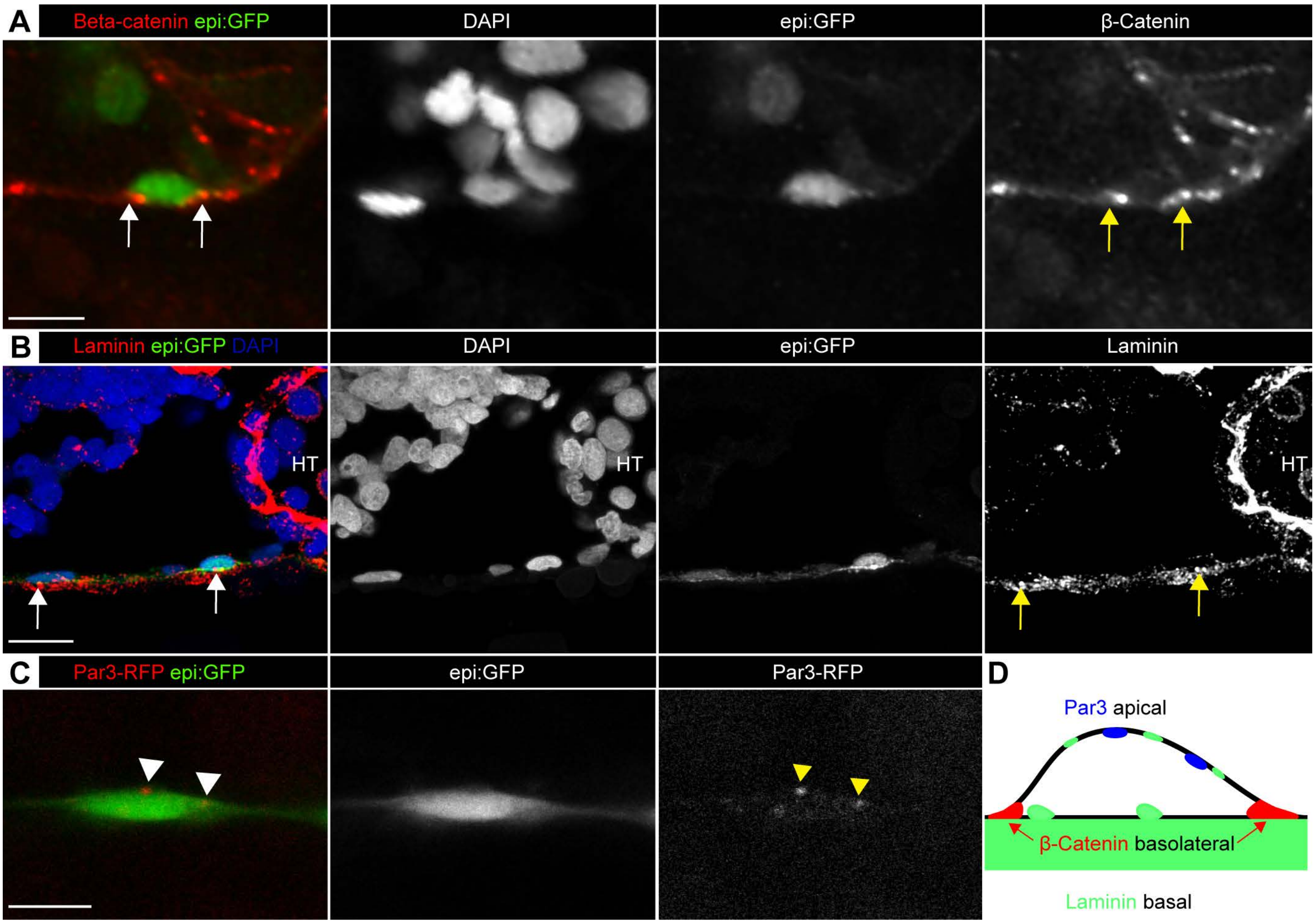

Fig. S2. Apicobasal polarity of dorsal pericardial cells. The figure shows examples of dorsal pericardial (DP) cells in epi:GFP larva at 52 hpf with fluorescently labeled cellular structures, indicating the apicobasal polarity. (A) Single optical 2 µm section of whole mount immuno-stained embryos (n=5 imaged embryos) against β-Catenin and GFP, nuclei were counterstained with DAPI. Arrows mark accumulation of β-Catenin at basolateral membranes of a DP cell. (B) Immunostaining on paraffin sections (n=3 imaged embryos) against Laminin and GFP, nuclei were counterstained with DAPI. Deconvolution was performed to increase the resolution. Shown is a maximum projection of 6.48 µm. Arrows point at Laminin accumulation on the basal side of the DP cell. (C) *In vivo* imaging of *Par3-RFP* (red) mRNA microinjected *epi:GFP* (green) embryos (n=3 imaged embryos). Arrowheads marks Par3-RFP signal on the apical side of the DP cell. Scale bars, 10 µm (5 µm in C). (D) Schematic representation of obtained results. Shown are representative images from ≥ 3 biological and ≥ 2 technical replicates.

Development • Supplementary information

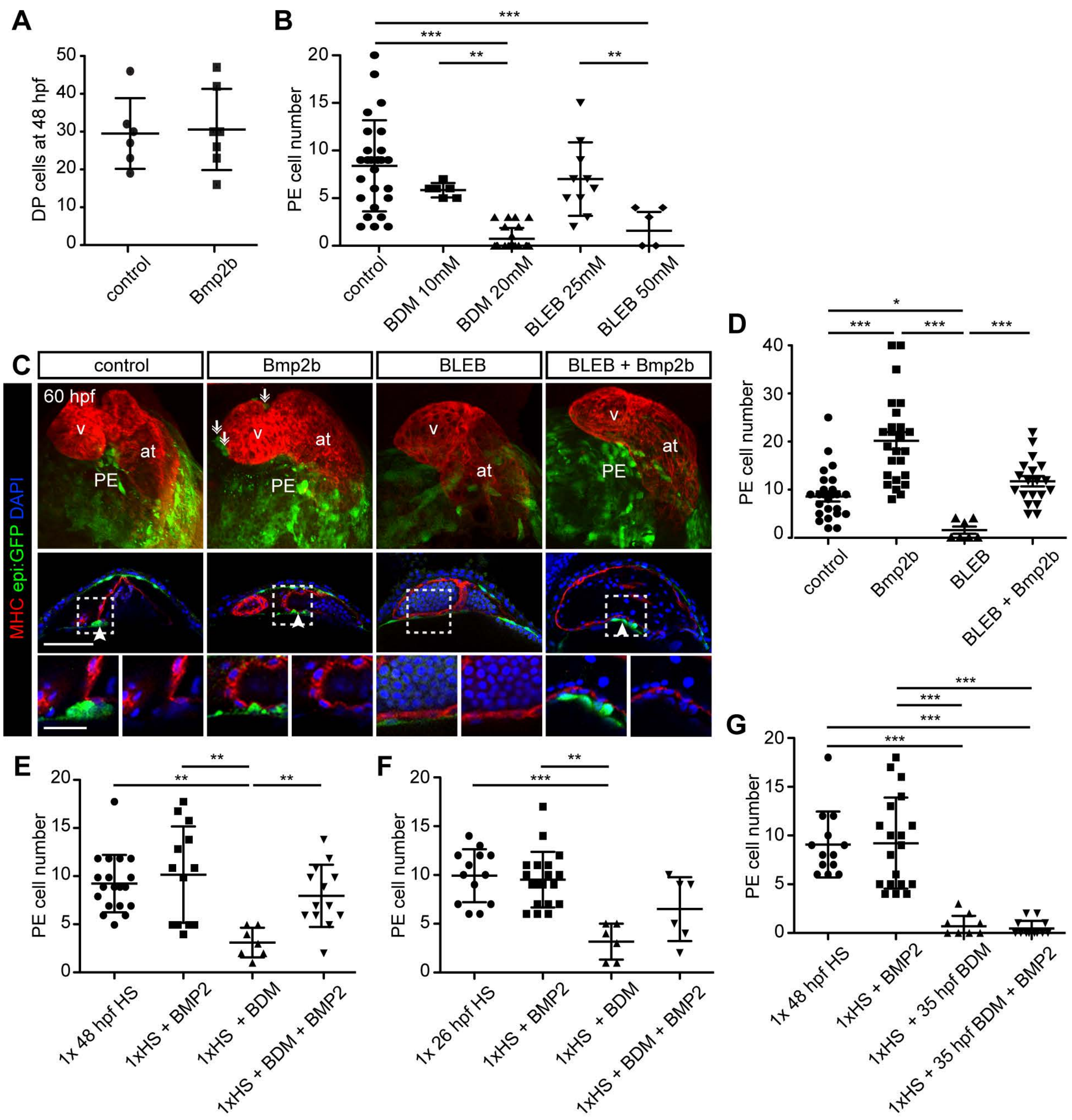

Fig. S3. *bmp2b* overexpression rescues PE formation in blebbistatin-treated fish. (**A**) DP total cell number at 48 hpf. (**B**) Quantification of PE cell number under different myosin II inhibitors (butanedione monoxime [BDM] or blebbistatin [BLEB]. (**C**) Maximal projections, optical sections and zoomed images (bottom panels) of 60 hpf *epi:GFP* embryos overexpressing *Bmp2b* with or without 50 µM BLEB. Embryos were immunostained for GFP (green) and myosin heavy chain (MHC, red). Nuclei were counterstained with DAPI (blue). Arrowheads indicate the PE. (**D**) Quantification of PE cell number from conditions shown in **C**. (**E-G**) Quantification of PE cell number under different conditions of BDM administration and *Bmp2* expression. (**E**) Quantification of PE cell number upon one heat shock (HS) at 48 hpf in non-transgenic or *hsp70:bmp2b* animals, with or without BDM treatment. (**F**) One HS at 26 hpf in non-transgenic or *hsp70:bmp2b* animals, with or without BDM treatment started at 48 hpf. (**G**) BDM-treatment started at 35 hpf, and *Bmp2b* overexpression at 48 hpf. at, atrium; DP, dorsal pericardium; hpf; hours post fertilization; HS heat shock; PE, proepicardium; v, ventricle;. Scale bar, 50 µm. In graphs, data are mean ± s.d. according to one-way ANOVA followed by Kruskal-Wallis test; * $P < 0.05$, **$P < 0.01$, *** $P < 0.001$. In **A** an unpaired two-tailed Student's *t*-test was used. Shown are representative images from ≥ 3 biological and ≥ 2 technical replicates.

Development • Supplementary information

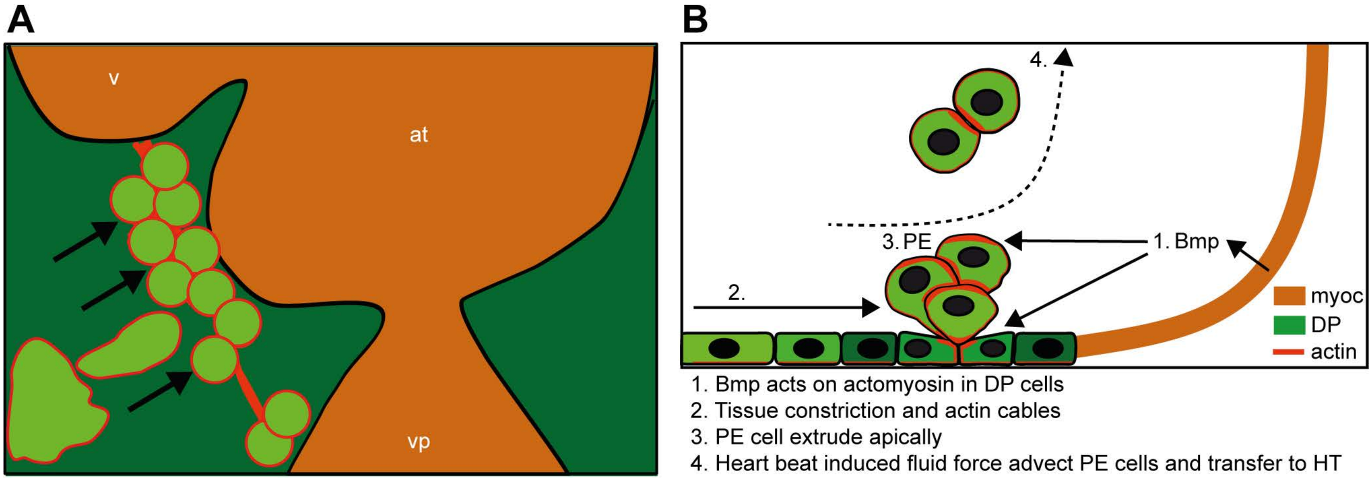

Fig. S4. Model of proepicardium formation. (A) DP cells displace towards the midline, where actin cables are visible, and reduce their cell area until rounding up into PE cells. **(B)** 1) Bmp acts on actomyosin in DP cells. 2) DP cells converge at the midline. 3) A local overcrowding and cell movements cause PE cells to extrude apically. 4) Ultimately, pericardial fluid flow forces evoked by the heartbeat wash away the PE cells, but the heartbeat is not needed for PE cells to extrude from the DP. at, atrium; DP, dorsal pericardium; PE, proepicardium; v, ventricle.

Development • Supplementary information

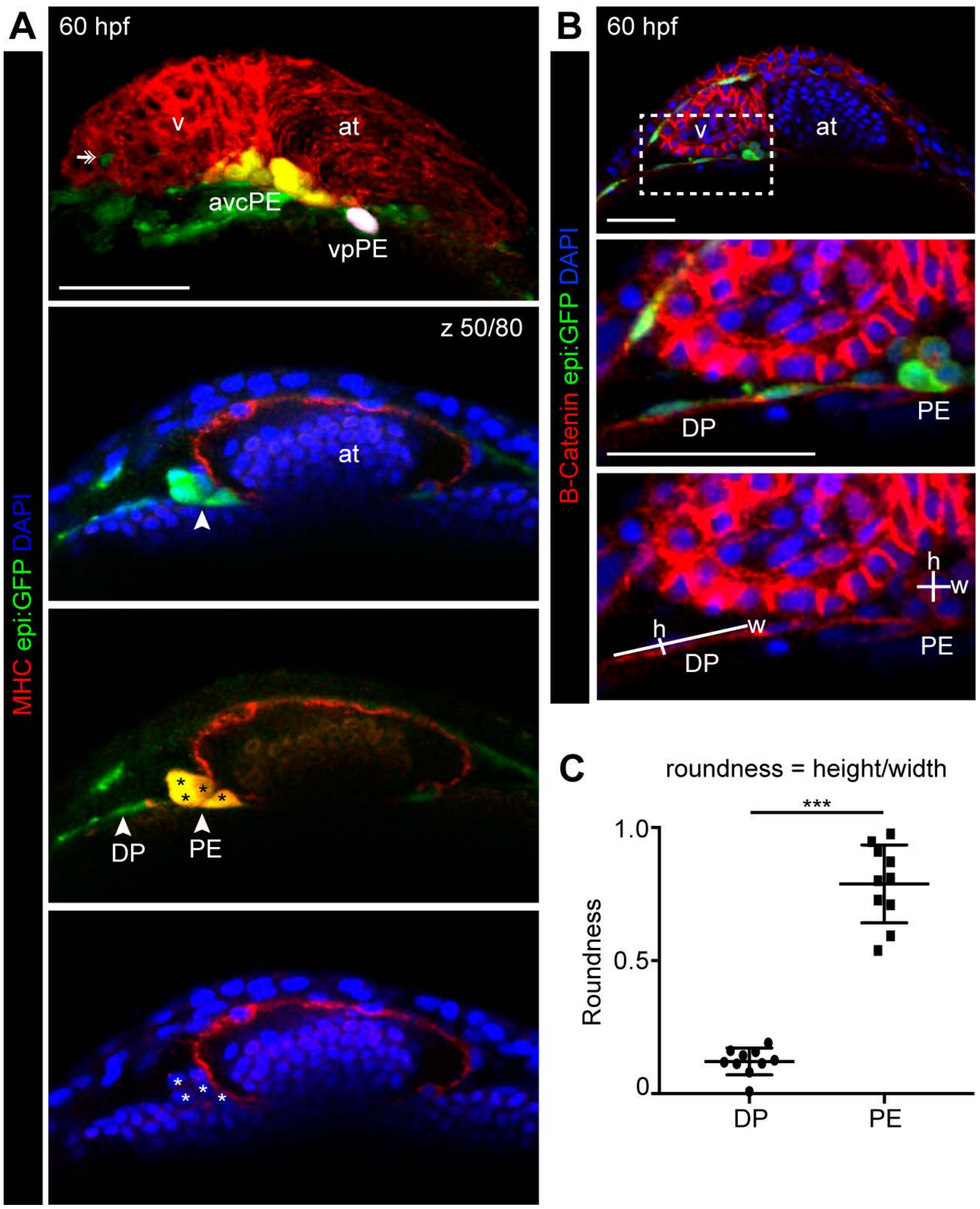

Fig. S5. Segmentation of PE from DP cells. (A) 3D top view of the heart region in a representative *epi:GFP* transgenic embryo immunostained for GFP (green), myosin heavy chain (MHC, myocardium) in red and counterstained with DAPI (blue). The ventral pericardium was digitally removed using Imaris to allow visualization of the underlying heart tube and DP. *epi:GFP*$^{+}$ cells in the DP and PE are shown in green, and different PE were segmented in Imaris showing avcPE in yellow and vpPE in white. From a z-stack of 80 slices taken every 4 µm, slice 50 is shown. White arrowheads point to a DP cell or PE cluster. Asterisks mark 4 PE cells (see also Movie 18). (**B**) Immunostaining of β-catenin was performed on *epi:GFP* embryos and nuclei were counterstained with DAPI. Overview and zooms show a single optical section. β-catenin marks the cell border and was used to identify the outline of cells. In the zoom white lines show the height (h) and width (w) of DP and PE cells. (**C**) The graph shows the roundness of DP and PE cells, the closer to one, the rounder the cell. at, atrium; DP, dorsal pericardium; hpf; hours post fertilization; PE, proepicardium; v, ventricle. Scale bar, 50 µm. Unpaired two-tailed Student's *t*-test was used. *** $P < 0.001$. Shown are representative images from ≥ 3 biological and ≥ 2 technical replicates.

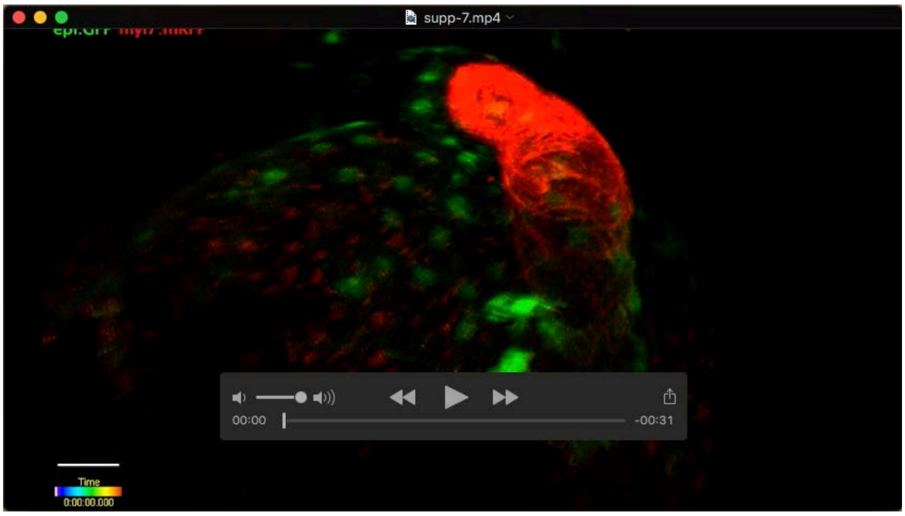

Movie 1. Tracking of dorsal pericardial cells. 3D reconstruction of an *in vivo* time-lapse imaging of *epi:GFP; myl7:mRFP* 52 hpf embryo during 18 h. Shown is a ventral view. GFP labels dorsal pericardium (DP) and proepicardium (PE) (green) and mCherry labels the myocardium of the heart tube in red. From frame 6 onwards, the DP was isolated and shown in a top 2D view. Blue dots indicate tracked DP cells, overall tracks are shown in frame 62 and 92; in frame 68, white arrows indicate the direction of the movement of tracked cells. Scale bar: 50 µm. Shown is a representative movie from ≥3 biological and ≥ 2 technical replicates.

Development • Supplementary information

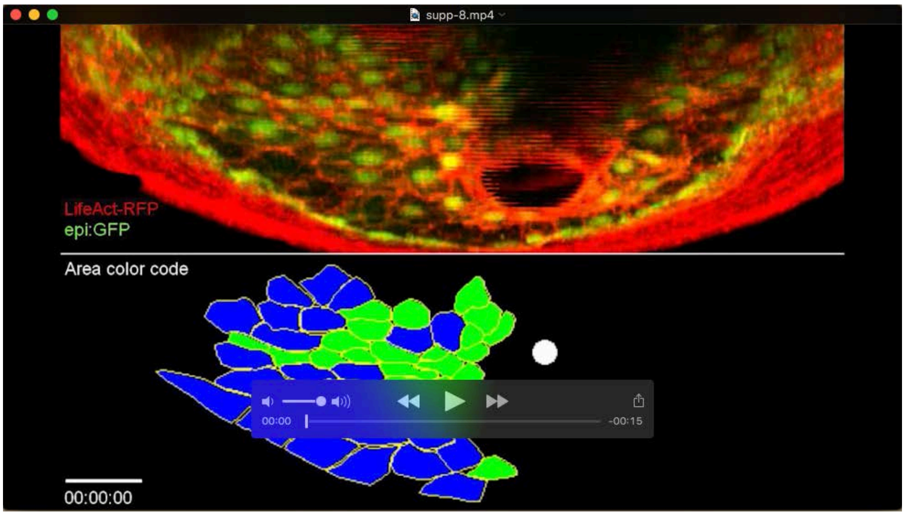

Movie 2. DP cell segmentation and area threshold color-map. Combined movie from a time-lapse using *βactin:LifeAct-RFP*^{e2212Tg};*epi:GFP* embryos from 48 hpf onwards and color coded segmentation of 47 cell shapes from time series above. White spots indicate reference location on the midline. Color-code: red, 0–50 µm^2; green, 50–150 µm^2; blue, >250 µm^2. Scale bar: 50 µm. Shown is a representative movie from ≥ 3 biological and ≥ 2 technical replicates.

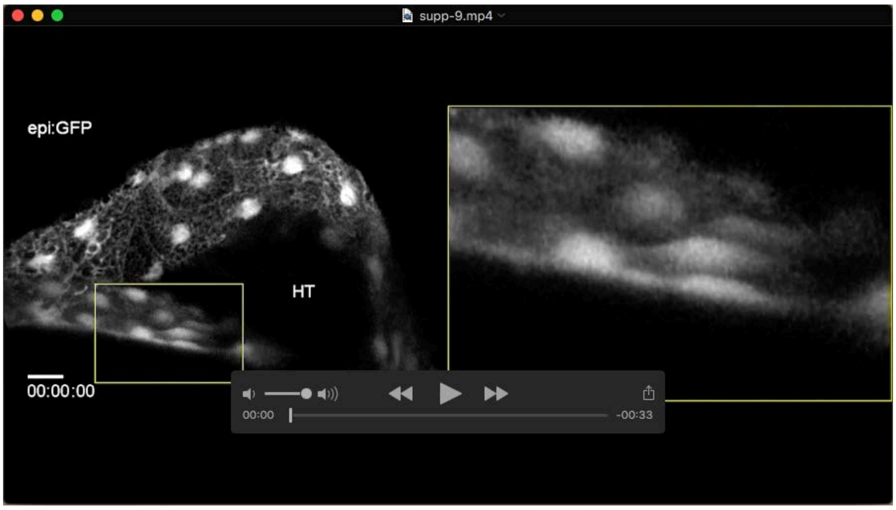

Movie 3. Convergence of dorsal pericardial cells leads proepicardial cells to extrude apically. *In vivo* time-lapse of *epi:GFP* embryo during 16.5 h starting at 52 hpf. The movie shows a maximum intensity projection of 22 µm. The left panel shows an overview of the ventral and dorsal pericardium (DP). Right panels show a zoomed frame of the time-lapse. An extruding PE cells can be observed. This movie is representative for the event observed in a total of 10 different movies. Scale bar of overview 20 µm and zoom 10 µm. HT, heart tube. Shown is a representative movie from ≥ 3 biological and ≥ 2 technical replicates.

Development • Supplementary information

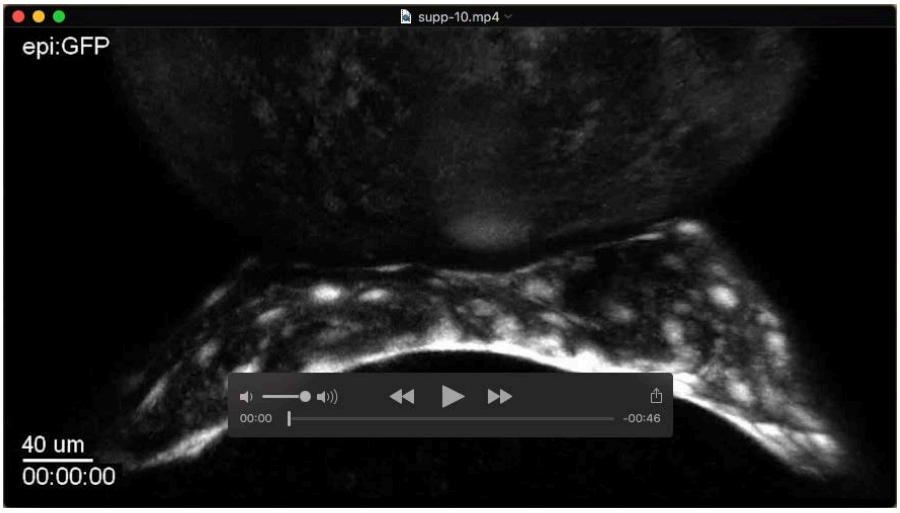

Movie 4. Cell divisions in the dorsal pericardium. 3D reconstruction of an *in vivo* time-lapse of an *epi:GFP* 35 hpf embryo during 33 h. GFP labels dorsal pericardial cells (DP, grey). The ventral pericardium was digitally extracted (white box) from frame 7 onwards to allow a top view on the DP. Ten cell divisions in the DP are indicated with yellow arrows. Shown is a representative movie from ≥ 3 biological and ≥ 2 technical replicates.

Development • Supplementary information

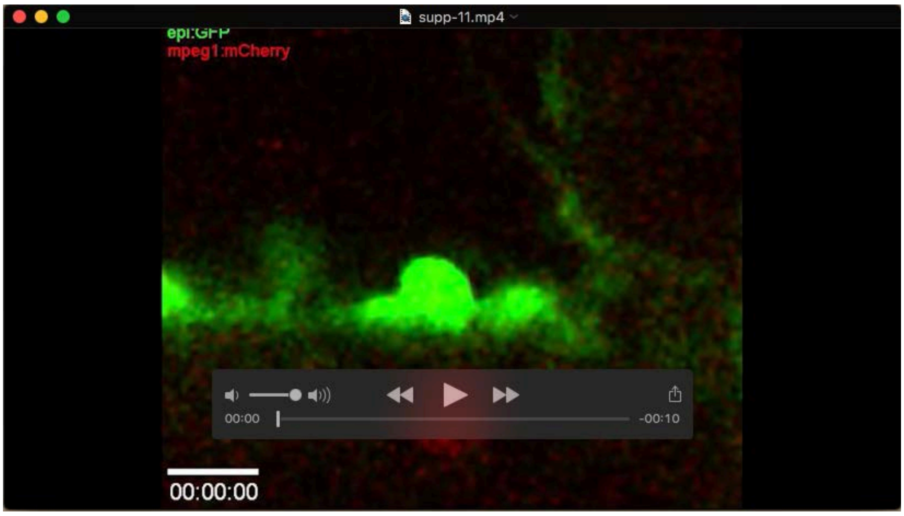

Movie 5. Macrophages remove emerging PE cells in the absence of a heartbeat. Movie of an *epi:GFP; mpeg1:mcherry* embryo microinjected with a *tnnt2* morpholino. One optical section is shown, after digitally fixing the extruded cell in the field of view. White arrowhead indicates extruding proepicardial (PE) cell (green) and magenta arrowhead shows a macrophage (red), which removes the PE cell by phagocytosis. Scale bar: 10 µm. Shown is a representative movie from ≥ 3 biological and ≥ 2 technical replicates.

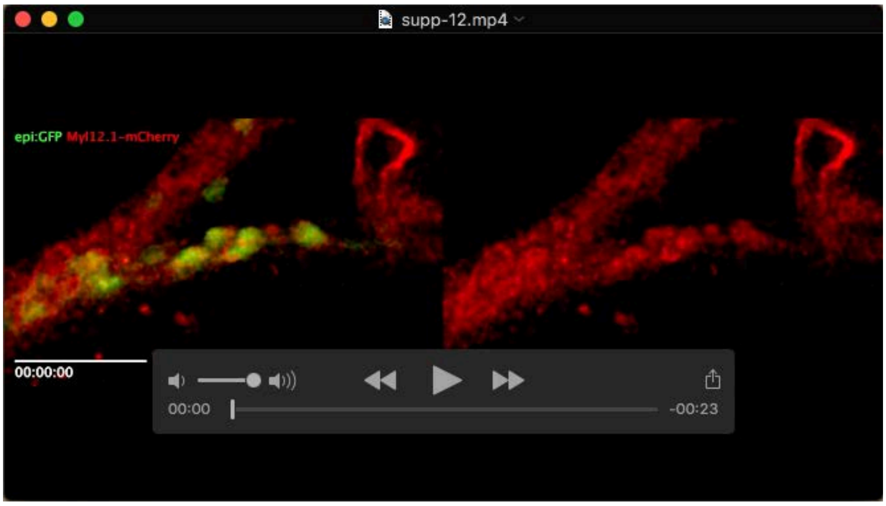

Movie 6. Myosin accumulates in proepicardial cells. *In vivo* time-lapse imaging of *epi:*GFP;*myl12.1:mCherry* 52-hpf embryo during 7 h. Optical section zoomed view of the proepicardial (PE) region. GFP labels dorsal pericardial (DP) cells (green). myosin12.1-mCherry is shown in red. Note the presence of myosin-positive PE cells and PE cells that are advected in the pericardial cavity. Scale bar: 50 µm. Shown is a representative movie from ≥ 3 biological and ≥ 2 technical replicates.

Development • Supplementary information

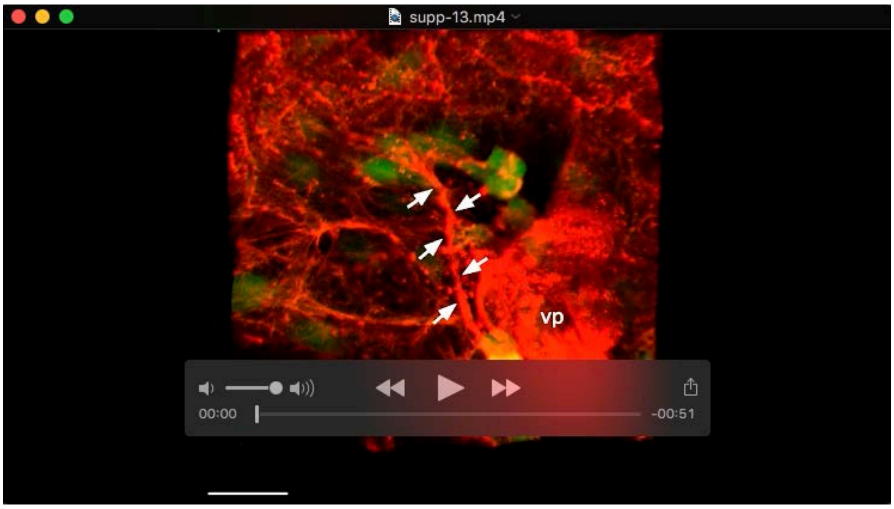

Movie 7. Actin cables in the dorsal pericardium. 3D reconstruction of an *in vivo* high resolution Z-stack of an *epi:GFP;β-actin:LifeAct-RFP* embryo at 52 hpf. GFP labels dorsal pericardial (DP) and proepicardial (PE) cells in green. LifeAct-RFP marks F-actin in red. The ventral pericardium was partially removed with a clipping plane in Imaris. An actin cable (arrows) connects DP cells together and with the venous pole (VP) of the heart tube. Scale bar: 10 µm. Shown is a representative movie from ≥ 3 biological and ≥ 2 technical replicates.

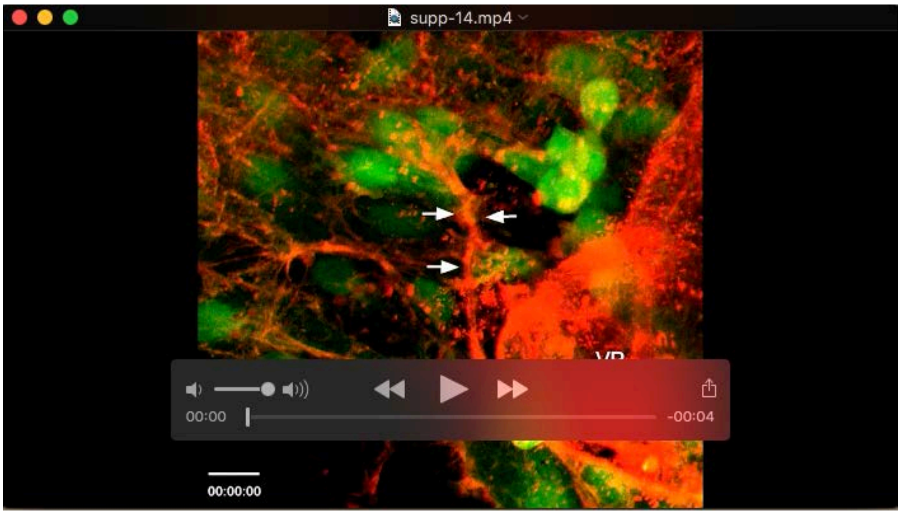

Movie 8. Actin cables in the dorsal pericardium. Maximum projection of the *in vivo* high resolution Z-stack shown in movie 7 from an *epi:GFP;β-actin:LifeAct-RFP* embryo at 52 hpf. GFP-labeled dorsal pericardium (DP) and proepicardial (PE) cells are shown in green. LifeAct-RFP marking F-actin is shown in red. Multiple actin cables are visible in the DP and one (arrows) connects a cluster of DP cells to the venous pole (VP) of the heart tube. This cluster joins a PE cluster closer to the VP and the midline at the end of the acquisition. During acquisition, the cable reduces in length and finally dissolves Scale bar, 20 µm. Shown is a representative movie from ≥ 3 biological and ≥ 2 technical replicates.

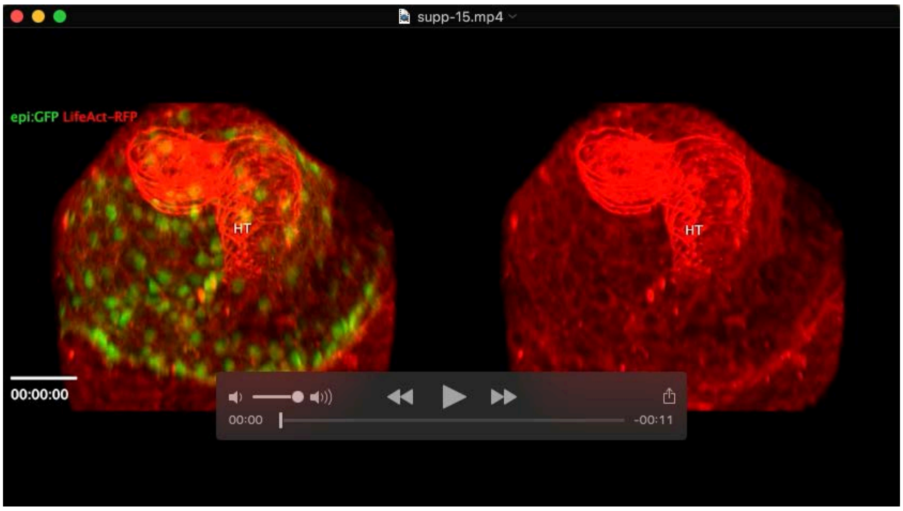

Movie 9. Actin accumulates at midline, where the PE forms. 3D reconstruction of an *in vivo* time-lapse imaging of an *epi:GFP;β-actin:LifeAct-RFP* 52-hpf embryo during 18.5 h. GFP-labeled dorsal pericardium (DP) and proepicardial (PE) cells are shown in green. LifeAct-RFP marking F-actin is shown in red. The ventral pericardium was digitally clipped out from frame 5 onwards to allow a better view on the DP. HT, heart tube. Scale bar: 50 µm. Shown is a representative movie from ≥ 3 biological and ≥ 2 technical replicates.

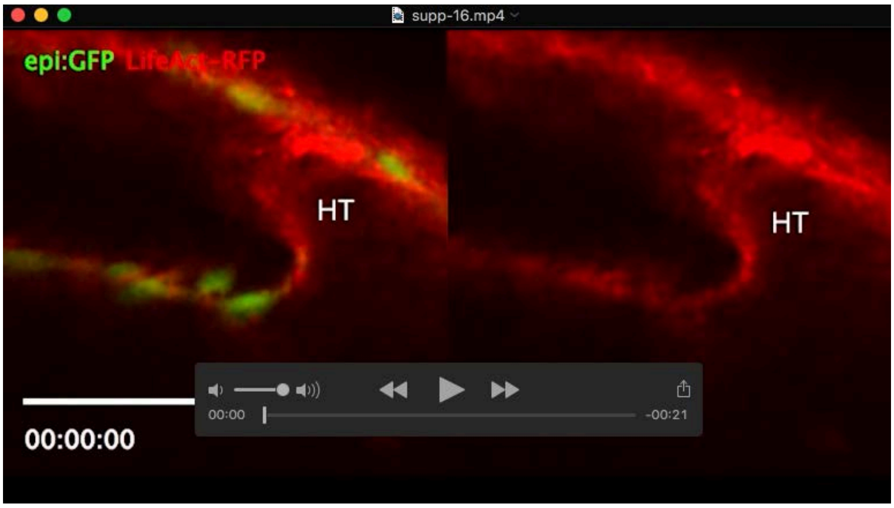

Movie 10. Proepicardial cells accumulate F-Actin. Optical section of 18 h *in vivo* time-lapse imaging of an *epi:GFP;β-actin:LifeAct-RFP* embryo starting at 52 hpf. GFP-labeled dorsal pericardium (DP) and proepicardial (PE) cells are shown in green. LifeAct-RFP marking F-actin is shown in red. Note that a subset of GFP^+ cells accumulate LifeAct-RFP and, at the end of the video, are released into the pericardial cavity (arrow). The remaining GFP-positve DP cells reveal lower LifeAct-RFP intensity. HT, heart tube. Scale bar: 50 µm. Shown is a representative movie from ≥3 biological and ≥ 2 technical replicates.

Development • Supplementary information

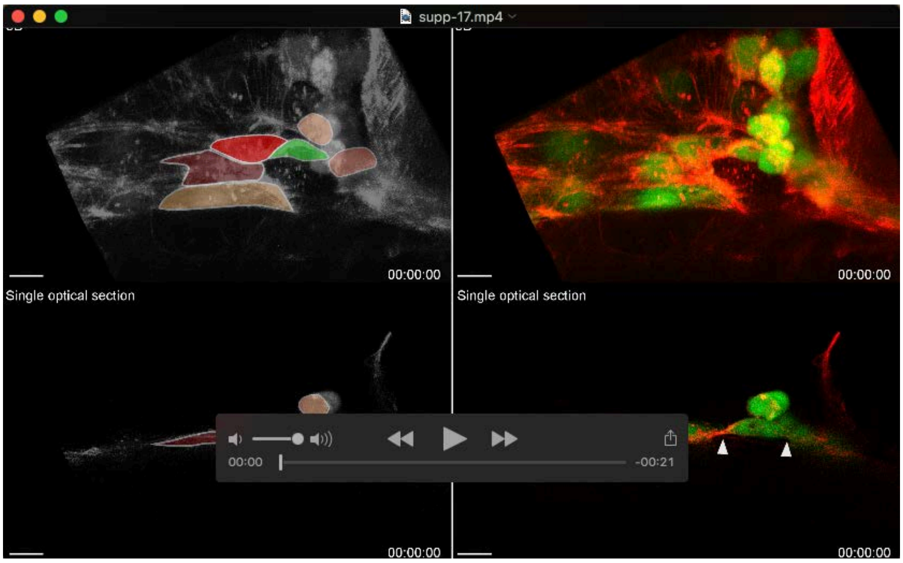

Movie 11. Apical extrusion pf PE cells. *epi:GFP;*β-*actin:LifeAct-RFP* embryo *in vivo* time-lapse starting at 52 hpf with drift correction. Top panels show 3D reconstruction, bottom panels show a single 0.320 µm thick optical section. On the right green (*epi:GFP)* labels dorsal pericardial (DP) and proepicardial (PE) cells in green. LifeAct-RFP marks F-actin in red. On the left, movie in gray-scale, the cell outlines were traced to create masks and each color identifies a cell over time. White arrowheads point to region were a cell extrusion will take place (cell labeled in green). At this site an actin ring becomes visible (red arrowhead). Scale bar, 10 µm. Shown is a representative movie from ≥ 3 biological and ≥ 2 technical replicates.

Development • Supplementary information

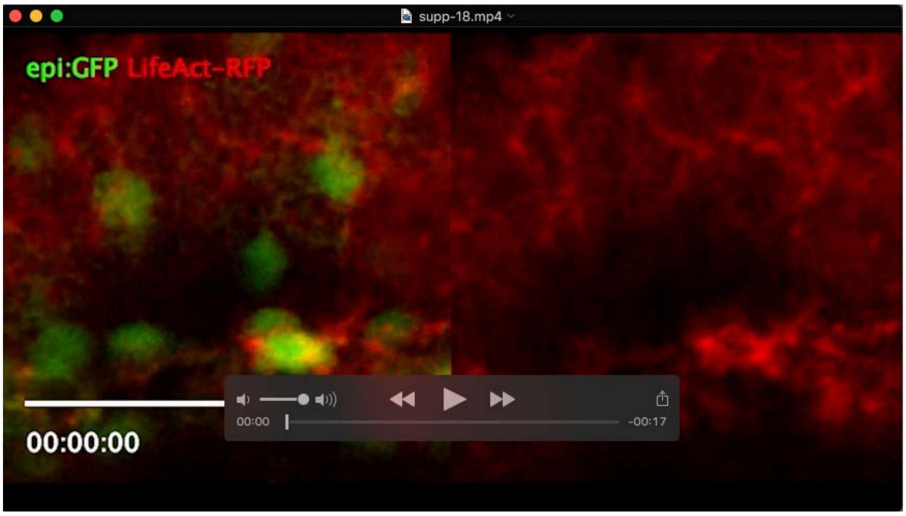

Movie 12. Proepicardial cells are attached to the dorsal pericardium by actin filopodia until release. Optical section of a 14 h *in vivo* time-lapse imaging of an *epi:GFP;β-actin:LifeAct-RFP* embryo starting at 52 hpf. GFP-labeled dorsal pericardium (DP) and proepicardial (PE) cells are shown in green. LifeAct-RFP marking F-actin is shown in red. Arrow in frame 75 points to an actin stalk connecting a PE cell with the DP. Scale bar: 50 µm. Shown is a representative movie from ≥ 3 biological and ≥ 2 technical replicates.

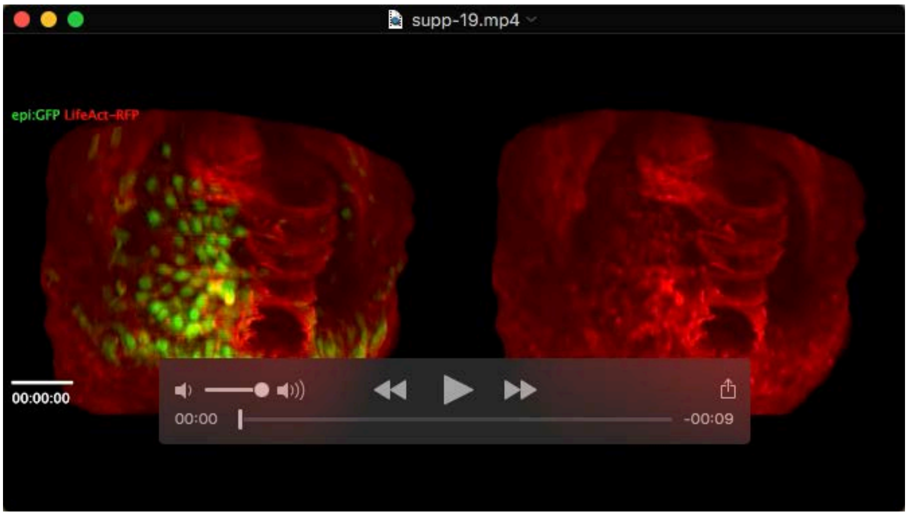

Movie 13. 2,3-Butanedione monoxime (BDM) inhibits F-actin accumulation at the dorsal pericardial midline. 3D reconstruction of *in vivo* time-lapse imaging of an *epi:GFP;β-actin:LifeAct-RFP* embryo starting at 52 hpf. The embryo was treated with 10 mM butanedione monoxime during acquisition. GFP-labeled dorsal pericardium (DP) and proepicardial (PE) cells are shown in green. LifeAct-RFP marking F-actin is shown in red. The ventral pericardium was digitally clipped out to allow a better view on the DP. On the left merged channels are shown. Scale bar: 50 µm. Shown is a representative movie from ≥ 3 biological and ≥ 2 technical replicates.

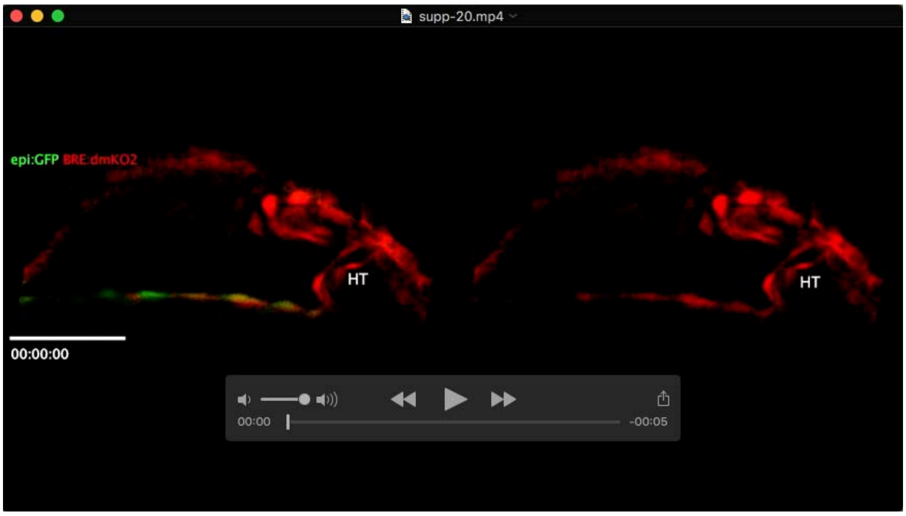

Movie 14. Bmp pathway acts transiently on proepicardial cells. Optical section (1 z-plane) of an *in vivo* time-lapse imaging of an *epi:GFP;BRE:KushabiraOrange* (KuO) 55 hpf embryo during 5.5 h. GFP-labeled dorsal pericardium (DP) and proepicardial (PE) cells (green) and cells active for the Bmp pathway are in red. Arrowhead in frames 15–22 points to a KuO^{+} PE cell. HT, heart tube. Scale bar: 50 µm. Shown is a representative movie from ≥ 3 biological and ≥ 2 technical replicates.

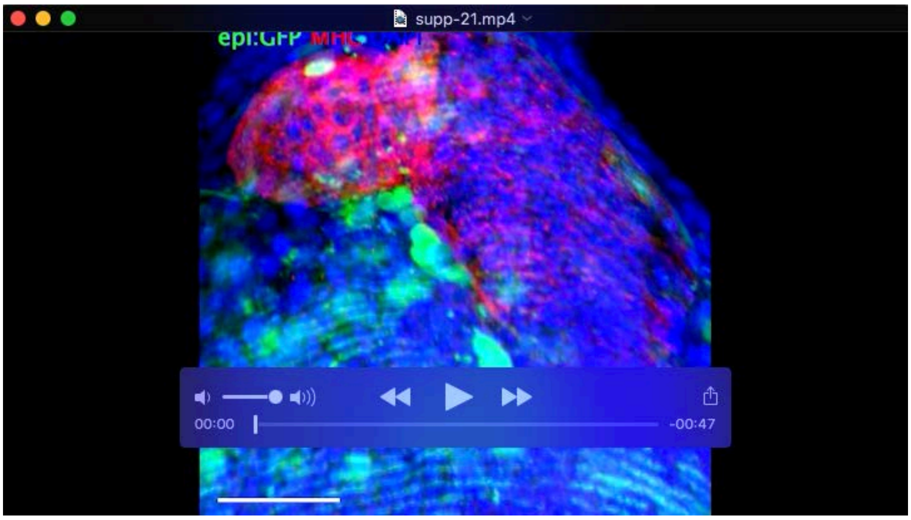

Movie 15. Segmentation of different PE clusters. 3D reconstruction of an *epi:*GFP 60 hpf zebrafish embryo. *epi:GFP* were immunostained for GFP in green, myosin heavy chain (MHC) in red and nuclei were counterstained with DAPI (blue). The ventral pericardium was digitally removed to allow a top view on the DP. avcPE and vpPE clusters were segmented (yellow and white colors, respectively). From second 10 onwards, transversal optical sections through the heart are shown to highlight the morphology of DP *vs* PE cells. at, atrium; avc, atrio-ventricular canal; DP, dorsal pericardium; PE, proepicardium; v, ventricle; vp, venous pole. Scale bar: 50 µm. Shown is a representative movie from ≥ 3 biological and ≥ 2 technical replicates.
